# Thermodynamic and Structural Characterization of a Mechanochemically Synthesized Pyrazinamide–Acetylsalicylic–Acid Eutectic Mixture

**DOI:** 10.3390/ph18020211

**Published:** 2025-02-05

**Authors:** Luís H. S. Queiroz, Mateus R. Lage, Clenilton C. dos Santos, Mafalda C. Sarraguça, Paulo R. S. Ribeiro

**Affiliations:** 1 Programa de Pós-Graduação em Ciência dos Materiais (PPGCM), Centro de Ciências de Imperatriz (CCIM), Universidade Federal do Maranhão (UFMA), Imperatriz 65900-410, Maranhão, Brazil; henrique_queiroz26@hotmail.com (L.H.S.Q.); mateus.lage@ufma.br (M.R.L.); 2 Laboratório de Espectroscopia Vibracional e Impedância (LEVI), Departamento de Física, Universidade Federal do Maranhão (UFMA), São Luís 65085-580, Maranhão, Brazil; cleniltoncs@gmail.com; 3 LAQV/REQUIMTE, Department of Chemical Sciences, Laboratory of Applied Chemistry, Faculty of Pharmacy, Porto University, 4050-313 Porto, Portugal; mafalda.cruz@ff.up.pt

**Keywords:** acetylsalicylic acid (ASA), pyrazinamide (PZA), drug–drug eutectic mixture (DDEM), DFT calculations, green chemistry, mechanochemistry, tuberculosis

## Abstract

**Background/Objectives:** This study aims to develop a sustainable and environmentally friendly drug delivery system by synthesizing a novel drug–drug eutectic mixture (DDEM) of acetylsalicylic acid (ASA) and pyrazinamide (PZA) using a green and efficient mechanochemical approach. **Methods:** The DDEM was characterized using various techniques, including differential scanning calorimetry (DSC), thermogravimetry and differential thermal analysis (TG-DTA), powder X-ray diffraction (PXRD), Fourier transform infrared spectroscopy (FT-IR), and Raman spectroscopy. Binary phase diagrams and Tammann’s triangle analysis determined the eutectic point. Density functional theory (DFT) calculations were performed on the starting compounds. The new system was evaluated for aqueous solubility, dissolution, and hygroscopicity. **Results:** A V-shaped binary phase diagram indicated the formation of a DDEM with a 2:1 molar ratio of ASA to PZA. A positive mixing enthalpy suggested a quasi-eutectic structure. The solubility of ASA and PZA increased by 61.5% and 85.8%, respectively, in the DDEM compared to the pure drugs. **Conclusions:** These findings highlight the potential of DDEMs to enhance drug properties and delivery. The synergistic interaction between ASA and PZA in the eutectic mixture may further improve therapeutic efficacy, warranting further investigation.

## 1. Introduction

Eutectic pharmaceutical systems have emerged as a promising approach to enhance the physicochemical properties of drugs [1,2]. A eutectic mixture (EM) is a combination of at least two solid components that undergo a phase transition into a liquid at a specific temperature, called the eutectic point. Substances in EMs are not isomorphous, and the components have a heterogeneous separation. These components are immiscible in the solid state, and there is no evidence of intermolecular interactions [3,4]. Unlike EMs, cocrystals, salts, and coamorphous systems exhibit intermolecular interactions, established between the drug and the coformer or another drug [5,6,7]. Drug–drug eutectic mixtures (DDEMs) are EMs composed of two or more drugs [2,8,9].

EMs can be prepared by various methods, including solvent evaporation, cooling, electrospray deposition, and mechanochemical techniques. Mechanochemical methods, such as neat grinding and liquid-assisted grinding (LAG), offer several advantages for the preparation of EMs. These methods are solvent-free or use minimal amounts of solvent, making them environmentally friendly and cost-effective [10]. Additionally, mechanochemical synthesis can be rapid, often requiring shorter reaction times compared to traditional solution-based methods [11]. This technique can also be applied to a wide range of compounds, including those with poor solubility, and can lead to the formation of metastable phases and amorphous materials, which can enhance drug dissolution and bioavailability. LAG involves the addition of a small volume of organic solvent to the solid starting materials. The milling process generates energy, promoting weak intermolecular interactions between the components. The solvent facilitates these interactions. After the reaction, the solvent evaporates completely, leaving no residual material. LAG offers advantages over neat grinding, such as reduced processing time and more efficient molecular interactions [2,12].

*Mycobacterium tuberculosis* (*M. tuberculosis*), the causative agent of tuberculosis (TB), a chronic infectious disease, continues to pose a significant global health threat. In 2021, it was the second deadliest infectious disease after SARS-CoV-2, with 10 million cases and over 1 million deaths [13,14]. Pyrazinamide (PZA), shown in Figure S1a, is an essential first-line anti-tuberculosis medication. It is used in conjunction with rifampicin, isoniazid, and ethambutol to treat tuberculosis, a combination often referred to as fixed-dose combinations [15,16]. This drug significantly shortens treatment duration by three months [14]; however, its efficacy is challenged by increasing resistance [17]. PZA, pyrazine-2-carboxamide, is a prodrug derived from nicotinamide and it is converted into its active pyrazinoic acid (POA) in an acidic environment [18]. The primary mechanism for POA (pKa = 2.9) [19] to exit the cell in *M. tuberculosis* is passive diffusion, with minimal contribution from efflux pumps. POA undergoes protonation to form HPOA, a molecule that readily crosses the plasma membrane. This can lead to cytoplasmic acidification, potentially inhibiting the activity of essential enzymes [16]. PZA is a water-soluble drug (15 mg.mL^−1^) with moderate permeability, classified as a BCS class III drug in the biopharmaceutical classification system [20]. This extremely weak base (pKa = 0.5) [19] has a melting range of 189–191 °C and exists in four polymorphic forms (α, β, γ, and δ) [21,22]. Form *α* is the most stable and commercially available form [20,23], while the other forms are metastable and transit to form α at room temperature.

Cocrystals of PZA have been reported with various acids, including 2,5-dihydroxybenzoic acid [24], p-aminobenzoic acid [25], p-toluenesulfonic, ferulic acid [26], oxalic acid [27], among others [28]. PZA has also formed cocrystals with other drugs, such as entacapone [29], diflunisal [30], pyrogallol [31], and theophylline [32]. Eutectic mixtures of PZA have been reported with saccharin, salicylic acid, cinnamic acid, nicotinic acid, isonicotinic acid [28], and isoniazid [33].

Acetylsalicylic acid (ASA), depicted in Figure S1b, also known as 2-acetyloxybenzoic acid, is a non-steroidal anti-inflammatory drug (NSAID). This weak acid has a reported pKa value of 3.50 and undergoes thermal decomposition after melting at approximately 140 °C [34,35]. ASA exhibits pH-dependent solubility characteristics, increasing as the pH rises above the p*K*a. It is classified as a BSC class I due to its high aqueous solubility (3.33 mg.mL^−1^) and membrane permeability [21,35]. ASA exists in three polymorphic forms: form I, form II, and form III [36]. Studies have explored the formulation of solid dispersions of ASA with other drugs and coformers [34,37,38,39,40,41].

Previous studies have shown that ASA can enhance the efficacy of PZA in reducing bacterial load [42] in TB infections [43,44]. Chen et al. [45] demonstrated that co-administration of weak acids, including salicylic acid, with PZA resulted in reduced bacterial loads of *M. tuberculosis* in vitro. Furthermore, Byrne et al. [42] showed that the combination of PZA with non-steroidal anti-inflammatory drugs (NSAIDs), such as ibuprofen and ASA, enhanced PZA’s potency in a mouse model of TB infection. In the mouse model, PZA alone reduced bacterial counts in the lungs from 6.9 ± 0.1 log10 CFU to 5.6 ± 0.5 log10 CFU. The combination of PZA with ASA further reduced lung bacterial counts to 5.0 ± 0.3 log10 CFU, representing an approximate 0.6 log10 CFU reduction compared to PZA alone. In the spleen, PZA alone reduced bacterial counts from 4.5 ± 0.2 log10 CFU to 2.7 ± 1.1 log10 CFU. While the ASA and PZA combination resulted in complete bacterial clearance in the spleen of two out of five mice, the overall reduction in spleen bacterial counts was not statistically significant compared to PZA alone. Additionally, PZA therapy can cause common side effects, such as nongouty polyarthralgias, which generally do not occur during the initial phase of treatment. Non-steroidal anti-inflammatory drugs, such as ASA, are generally effective in alleviating the pain associated with this adverse effect [46]. Current PZA formulations may have limitations, such as potential drug–drug interactions and gastrointestinal side effects, which can impact patient adherence and overall treatment outcomes. The emergence of drug-resistant strains of *M. tuberculosis* further underscores the need for improved treatment strategies. These findings, coupled with the potential to mitigate PZA-related side effects, suggest that the combination of ASA and PZA in a drug delivery system, such as a DDEM, could significantly enhance the therapeutic efficacy of both drugs, particularly in the treatment of drug-resistant TB.

This study employed the green and efficient liquid-assisted LAG technique to prepare a novel DDEM of PZA and ASA. The eutectic composition was determined using binary phase diagrams and Tammann’s triangle analysis. A comprehensive solid-state characterization, including differential scanning calorimetry (DSC), thermogravimetry and differential thermal analysis (TG-DTA), powder X-ray diffraction (PXRD), Fourier transform infrared spectroscopy (FT-IR), and Raman spectroscopy, was performed. To understand molecular interactions and the energy landscape, density functional theory (DFT) calculations were performed. Additionally, the aqueous solubility and dissolution profiles of the DDEM were determined.

## 2. Results and Discussion

### 2.1. Thermal Analysis

**Figure 1:**
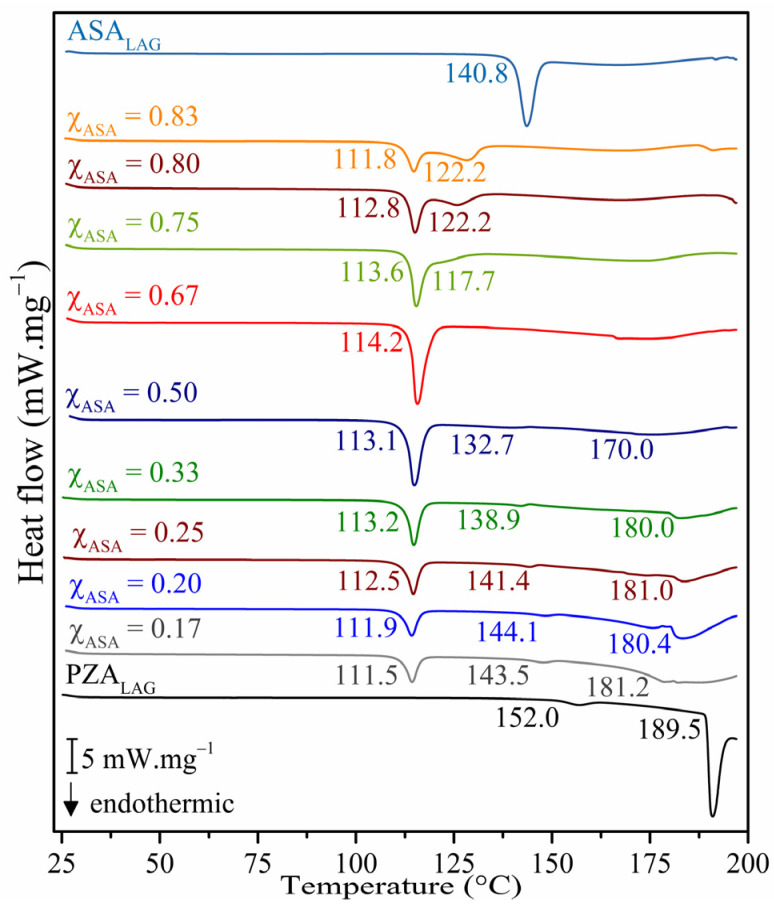
DSC curves of ASA_LAG_, PZA_LAG_, and mixtures prepared by the liquid-assisted grinding (LAG) method, with 0.17, 0.20, 0.25, 0.33, 0.50, 0.67, 0.75, 0.80, and 0.83 molar ratios of ASA.

DSC was used to study the eutectic composition [47]. ASA_LAG_, PZA_LAG_, and mixtures prepared by the LAG method with molar ratios of ASA ranging from 0.17 to 0.83 were analyzed. Figure 1 illustrates the resulting DSC curves, while Table S1 (Supporting Information) summarizes the key thermal events.

The DSC thermograms of ASA and PZA revealed the following: ASA exhibits a melting point of 140.8 °C, with an enthalpy of fusion (ΔH*_f__us_*) of 41.33 kJ.mol^−1^ [37]. PZA underwent a solid–solid phase transition (form α → form γ) at 152.0 °C with a transition enthalpy (ΔH*_trs_*) of 1.52 kJ.mol^−1^, followed by a melting point at 189.5 °C (ΔH*_fus_* = 26.69 kJ.mol^−1^) [22]. The physical mixture (PM) displayed a melting point of 115.3 °C (Figure S2, Supporting Information).

The phase diagram plots the melting temperature against the mole fraction of one component. The solidus point represents the T*_fus_* of the eutectic mixture, while the liquidus point corresponds to the excess component. The V-shape pattern in the diagram indicates the eutectic point, characterized by its minimum melting temperature and specific molar ratio [48]. The ΔH*_fus_* was used to determine the eutectic stoichiometry across the range of mixtures. As the relative proportions of the components vary, the ΔH*_fus_* value increases to its maximum at the eutectic composition and then decreases. This maximum value corresponds to the eutectic point, indicating the optimal composition for the lowest melting point [2,49].

A binary phase diagram and a Tammann’s triangle were constructed using the mole fraction of ASA as the independent variable. The eutectic point was determined from these graphical representations. The experimental data used to construct these diagrams are presented in Table S2. The DSC curves for ASA-PZA mixtures (Figure 1) exhibited a common melting point range of 111–114 °C. The congruent melt in the phase diagram and the inflection point in Tammann’s triangle suggest a eutectic stoichiometry of 2:1 (χ_ASA_ = 0.67). This stoichiometry is further supported by the V-shape of the phase diagram, which arises from the significantly lower melting point of the eutectic mixture (T*_fus_* = 114.2 °C) compared to the pure components (ASA_LAG_: T*_fus_* = 140.8 °C; PZA_LAG_: T*_fus_* = 189.5 °C). Based on these findings, the ASA-PZA (2:1) mixture (DDEM) was selected for further characterization and evaluation.

**Figure 2:**
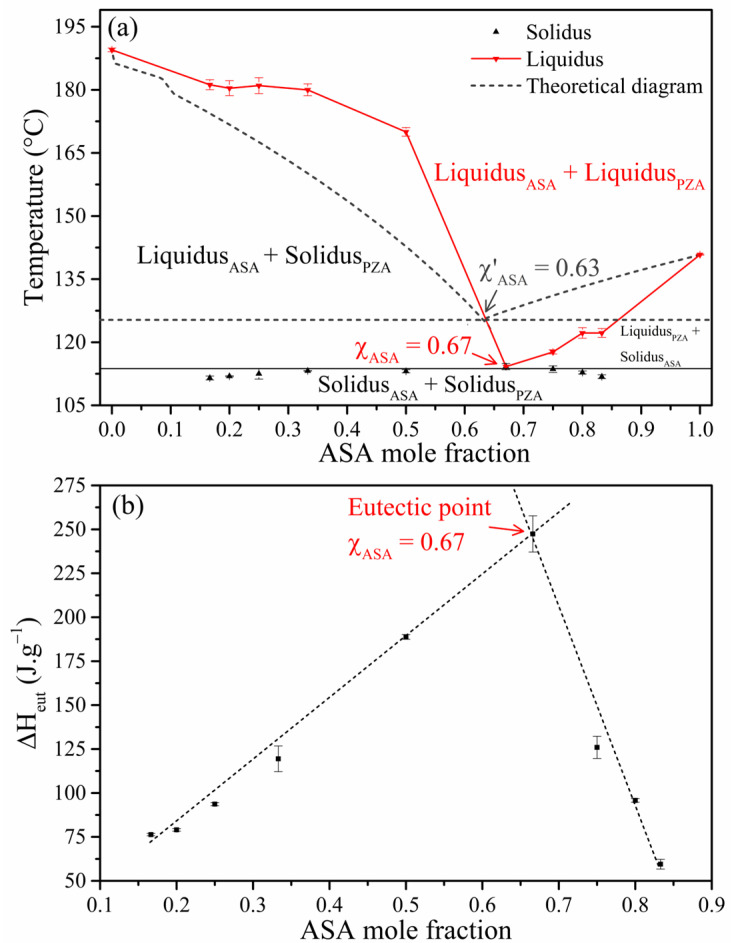
Theorical and experimental binary diagram (**a**) and Tammann’s triangle (**b**) of ASA, PZA, and mixtures prepared by the LAG method with ASA molar ratios of 0.17, 0.20, 0.25, 0.33, 0.50, 0.67, 0.75, 0.80, and 0.83.

The theoretical phase diagram, constructed using the values in Table S3 (Supporting Information), predicted the formation of a eutectic mixture at χ’_ASA_ = 0.63 (Figure 2). The slight discrepancy between the theoretical and experimental eutectic compositions can be attributed to factors such as limitations in the theoretical model and the influence of intermolecular interactions.

The nondimensional entropy of fusion (ΔS*_fus_*^0^) for pure components and DDEM are presented in Table 1. The entropy of fusion (ΔS*_fus_*) was calculated using the enthalpy of fusion and melting temperature [8,50].

Simple binary eutectic systems can be classified based on the entropies of fusion of their individual components. If one component has a low entropy of fusion (ΔS*_fus_*^0^ < 2), the eutectic crystallization exhibits “couple growth”, where both components grow simultaneously in a coordinated manner, resulting in a well-defined microstructure. Conversely, if both components have high entropies of fusion, they grow independently, forming a simple physical mixture of anisotropic/faceted crystals [51].

The starting materials ASA_LAG_ and PZA_LAG_ have ΔS*_fus_*^0^ values greater than 2, indicating that the resulting compound is a simple mixture. The DDEM also has ΔS*_fus_*^0^ > 2, suggesting a higher nondimensional entropy of fusion [52]. This implies that the ΔS*_fus_* of the DDEM is greater than that of the crystalline ASA and PZA. This elevated thermodynamic state of the DDEM may be attributed to significant micronization [8], a process that reduces particle size and increases the surface area of a substance. This increased surface area can lead to enhanced molecular mobility and weaker intermolecular interactions within the DDEM, resulting in a higher entropy of fusion. This higher entropy state can contribute to the formation of a more disordered, quasi-eutectic structure.

Thermochemical studies indicate that the structural arrangement within a eutectic melt is dependent on the sign and magnitude of the mixing enthalpy. Three possible structures include quasieutectic ($\Delta^{M}H$ > 0), clustering of molecules ($\Delta^{M}H$ < 0), and molecular solutions ($\Delta^{M}H$ = 0) [53,54]. For the ASA-PZA system, the experimentally determined mixing enthalpy (${(\Delta }_{fus}{H^{0})}_{exp}$) was 119.21 kJ.mol^−1^, while the calculated value (${(\Delta }_{fus}{H^{0})}_{calc}$) was 37.60 kJ.mol^−1^. The positive difference that corresponds to the value of the mixing enthalpy ($\Delta^{M}H$ = 81.61 kJ.mol^−1^), which indicates weak intermolecular interactions between the components in the melt, is indicative of a quasi-eutectic structure in the binary eutectic melt.

The TG/DTG-DTA curves of ASA_LAG_, PZA_LAG_, DDEM, and PM are presented in Figure S3 (Supporting Information), while the corresponding thermal parameters are summarized in Tables S4 and S5 (Supporting Information).

This technique provides insights into the thermal stability and decomposition behavior of the samples. ASA_LAG_ exhibits thermal stability up to 135 °C, followed by a two-stage decomposition process: 135–257 °C (mass loss, Δ*m* = 49.5%) and 257–360 °C (Δ*m* = 47.7%). The DTA reveals a sharp endothermic peak at 139.0 °C, corresponding to the melting point (ΔH*_fus_* = 204.33 kJ.mol^−1^). The two additional endothermic peaks at 163.5 °C and 346.6 °C correspond to the decomposition processes [40]. PZA_LAG_ is thermally stable up to 134 °C, followed by sublimation (134–188 °C, Δ*m* = 30.5%) and evaporation (188–226 °C, Δ*m* = 67.9%) processes. The DTA curve shows a solid-solid phase transition (form α → form γ) at 152.1 °C (ΔH*_fus_* = 3.67 kJ.mol^−1^), a melting point at 188.6 °C (ΔH*_fus_* = 80.96 kJ.mol^−1^), and a final decomposition step at 203.5 °C [22]. The DDEM is thermally stable up to 134 °C where it decomposes in two stages: 134–252 °C (Δ*m* = 54.1%) and 252–363 °C (Δ*m* = 42.4%). The DTA curve shows a low melting point at 113.6 °C (ΔH*_fus_* = 571.76 kJ.mol^−1^) followed by decomposition at 152.1 °C and 294.8 °C. The PM exhibits similar thermal behavior, with a melting point at 115.9 °C. While the melting point decreased in the PM, the enthalpy of fusion increased (Figure S2, Table S1), indicating the effectiveness of the preparation method.

### 2.2. Structural Characterization and Molecular Interactions

PXRD is a powerful technique for distinguishing eutectic mixtures from cocrystals/salts. The absence of new diffraction peaks in the PXRD pattern of a eutectic mixture indicates the absence of a new crystalline phase, confirming the molecular distribution of the individual components within the crystal lattice. In contrast, cocrystals/salts formation leads to significant changes in the diffractogram profile [2].

FT-IR and Raman spectroscopy are complementary techniques that analyse molecular vibrations, providing insights into structure and interactions. However, these techniques have limitations in quantifying weak intermolecular interactions, which are crucial for a comprehensive understanding of the system. Therefore, a combination of PXRD and spectroscopic techniques is essential for elucidating the crystal lattice interactions [55].

**Figure 3:**
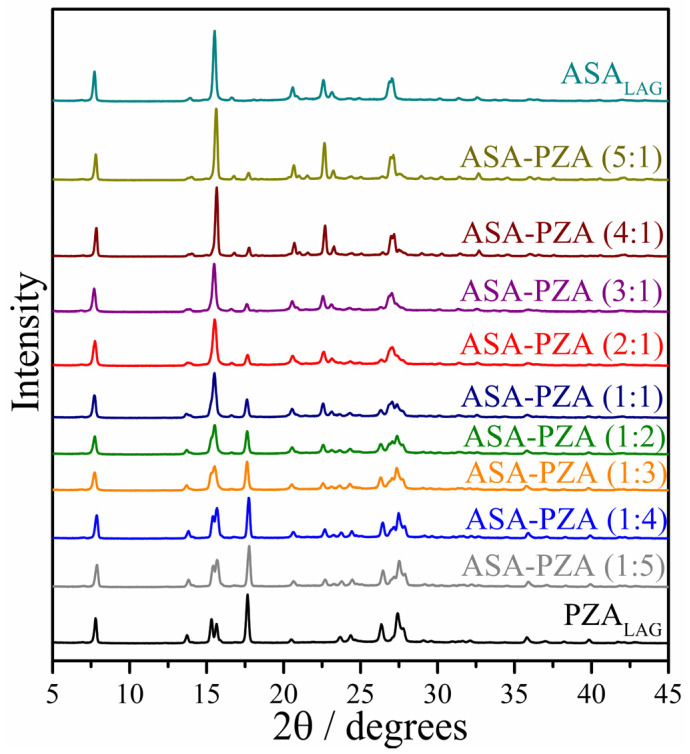
PXRD patterns of ASA_LAG_, PZA_LAG_ and ASA-PZA mixtures.

PXRD patterns of ASA_LAG_, PZA_LAG_ and various ASA-PZA molar ratios are shown in Figure 3. Comparison with CCDC diffractograms confirmed that grinding did not alter the polymorphic forms of ASA and PZA, as evidenced by the Rietveld refinement quality parameters (Figure S4, Supporting Information).

ASA_LAG_ shows PXRD peaks at 7.7°, 13.9°, 20.6°, 22.6°, 23.1° and 27.0° (2*θ*), consistent with form I (REFCODE: ACSALA29) [56]. Rietveld refinement yielded R_wp_ = 10.2% and S = 2.2. PZA_LAG_ exhibits peaks at 7.8°, 13.7°, 15. 3°, 15.6°, 17.6°, 20.5°, 23.7°, 24.4°, 26.3°, and 27.4° (2*θ*), corresponding to form α (REFCODE: PYRZIN) [57]. Its refinement resulted in R_wp_ = 9.8% and *S* = 1.9. Lattice parameters are summarized in Table S6 (Supporting Information).

The PXRD pattern of the DDEM shows the characteristics peaks of both ASA (form I) and PZA (form α) without peak shifts. Rietveld refinement (Figure S5, Supporting Information) confirms the presence of two crystalline phases within DDEM, with no evidence of detectable amounts of other crystalline phases. The absence of new peaks in the PXRD pattern indicates the absence of significant intermolecular interactions between the drug components, as expected in a eutectic mixture. The refinement accuracy of R_wp_ = 7.8% and *S* = 1.6 suggests a good fit between the experimental and calculated diffraction patterns.

**Figure 4:**
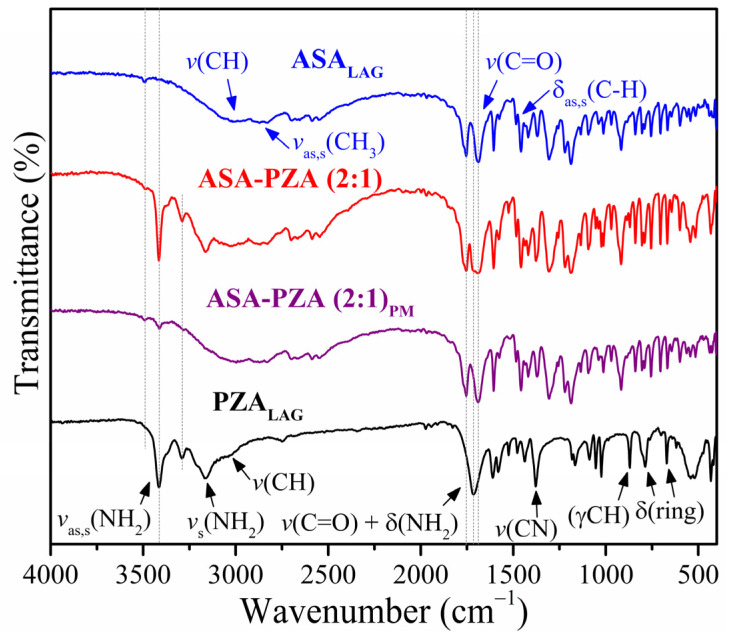
IR spectra of ground acetylsalicylic acid (ASA_LAG_), ground pyrazinamide (PZA_LAG_), DDEM (ASA-PZA (2:1)) and its PM (ASA-PZA (2:1)_PM_).

Figure 4 shows the infrared (IR) spectra of ASA_LAG_, PZA_LAG_, DDEM and PM. Table S7 (Supporting Information) summarizes the vibrational bands, which are characteristics of form I for ASA_LAG_ [40] and form α of PZA_LAG_ [58].

The IR spectra of ASA_LAG_ shows characteristic vibrations associated with the aromatic rings at 3040–3100 cm^−1^, while the -CH_3_ stretching vibrations appear at 2770–2850 cm^−1^. The bands at 2770–2850 cm^−1^ are due to the symmetrical and antisymmetrical stretching vibrations of the -CH_3_. Intense C=O bands are observed at 1754 and 1692 cm^−1^. The *v*C=C aromatic vibration occurs at 1605 cm^−1^, *v*C-O-H in-plane vibration at 1457 cm^−1^, and *v*O-H plane in-plane vibration at 1419 and 1371 cm^−1^. A strong δ_as,s_C-H strain vibration appears at 1303 cm^−1^, *v*C-O vibration at 1221 cm^−1^, and *v*OC=C vibration at 1188 cm^−1^. Out-of-plane C-H bending of the phenyl ring occurs at 917, 840, 803, and 754 cm^−1^, while the out-of-plane C-C bending occurs at 704 cm^−1^.

PZA_LAG_ shows NH_2_ stretching vibrations (symmetrical and antisymmetrical) at 3414 cm^−1^ and symmetrical vibrations a at 3364 (sh), 3211 (sh) and 3163 cm^−1^. The *v*(CH) vibration appears at 3086 cm^−1^, and the amide *v*(C=O) + δ(NH_2_) vibration at 1714 cm^−1^. Amide δ(NH_2_) in-plane bending occurs at 1611 cm^−1^, and pyrazine ring vibrations are observed at 1582, 1525, 1479, and 1438 cm^−1^. The *v*(C-N) vibration appears at 1379 cm^−1^, and other bands at lower wavenumbers correspond to pyrazine ring bending.

The IR spectra of the pure components and the eutectic mixture exhibited a high degree of similarity, with no significant shifts in peak positions. This observation suggests that the eutectic formation does not involve strong intermolecular interactions between the drug molecules. Instead, the components retain their individual molecular structures within the eutectic mixture.

**Figure 5:**
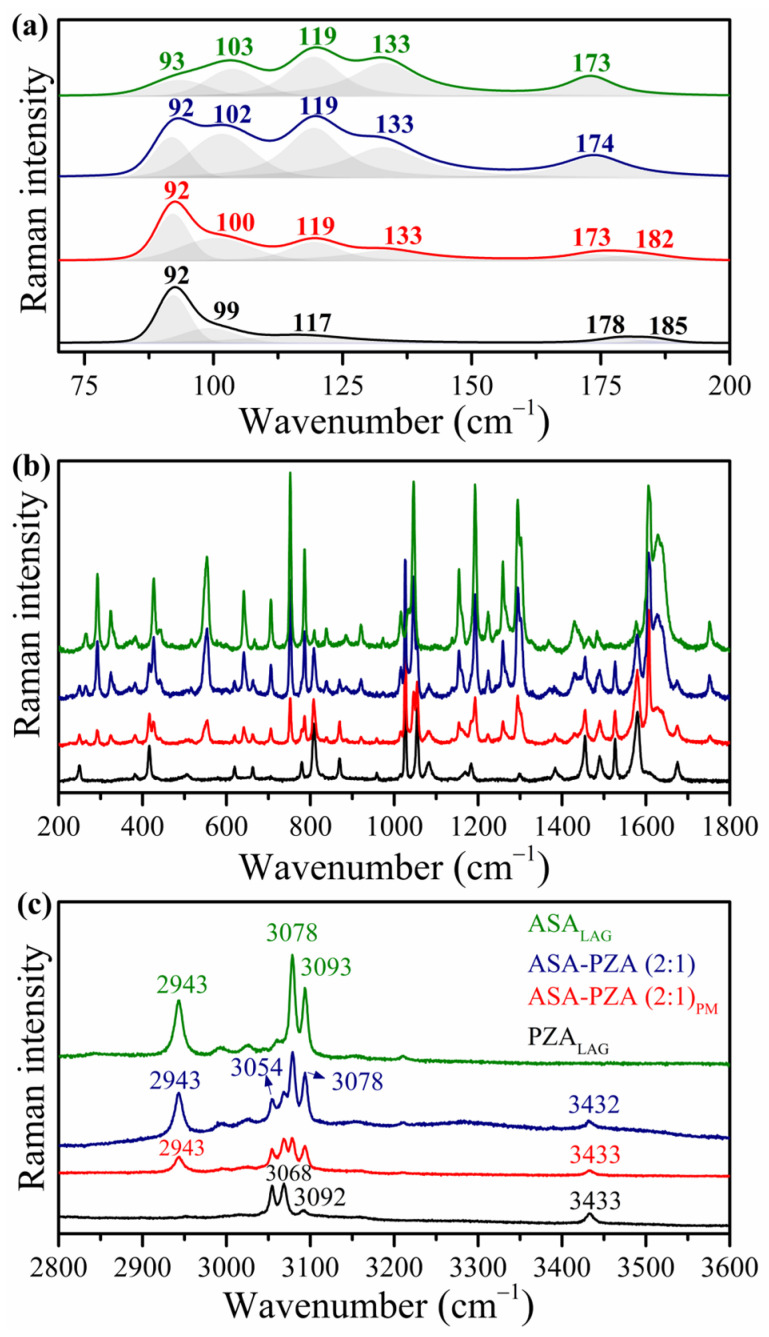
Raman spectra of ASA_LAG_ (green), PZA_LAG_ (black), DDEM (ASA-PZA (2:1)) (blue), and PM (ASA-PZA (2:1)_PM_) (red); Spectral region: (**a**) 70-200 cm^−1^; (**b**) 200-1800 cm^−1^; and (**c**) 2800-3600 cm^−1^.

Figure 5 shows the Raman spectra of ASA_LAG_, PZA_LAG_, DDEM, and PM (Table S8 in Supporting Information, which lists the corresponding wavenumbers) [36,59,60]. The Raman spectra of the DDEM closely resemble those of the individual components, with no significant shifts in peak positions. This observation further supports the formation of a simple eutectic mixture, where the components retain their individual molecular structures and weak intermolecular interactions. In contrast, the formation of cocrystals or salts would involve stronger intermolecular interactions and result in significant changes in the vibrational spectra [2].

### 2.3. Computational Studies

For an eutectic mixture to form, the components must be miscible in the liquid state but immiscible in the solid state. In this system, the components must have compatible functional groups that can form weak intermolecular interactions, such as hydrogen bonding, van der Waals forces, or dipole–dipole interaction [61,62,63]. These interactions can lower the melting point of the mixture and stabilize the eutectic phase.

To investigate the electronic structure and reactivity of ASA and PZA, density functional theory (DFT) calculations were performed. The geometries of ASA and PZA were optimized using the *ω*B97X-D functional and the 6-311++G(d,p) basis set in the gas phase. Frontier molecular orbitals (HOMO and LUMO) were calculated to determine reactivity descriptors such as chemical hardness (*η*), softness (*σ*), electronegativity (*χ*), chemical potential (*μ*), and global electrophilicity index (*ω*). These descriptors provide insights into the molecular reactivity and possible molecular interactions [61].

**Figure 6:**
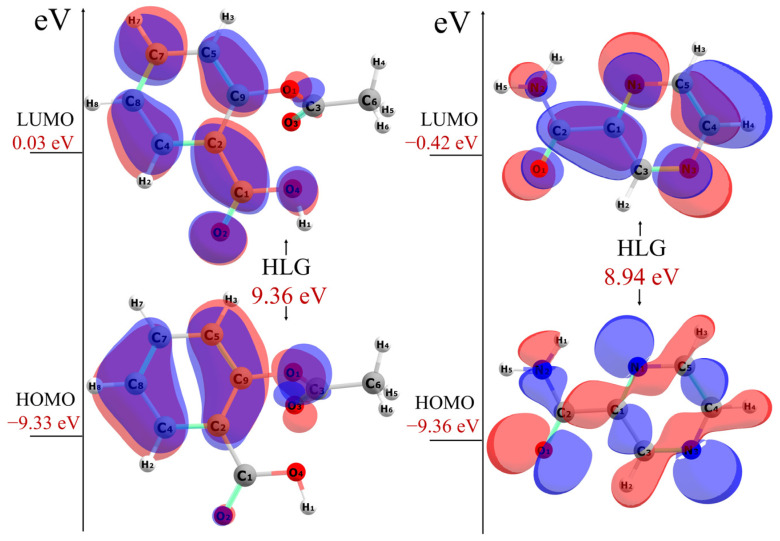
HOMO and LUMO for ASA (**left**) and PZA (**right**) in vacuum, obtained from calculations employing the DFT functional *ω*B97X-D and 6-311G++(d,p) basis set.

The HOMO is associated with electron-donating ability, and its negative energy corresponds to the ionization energy (IE). The LUMO is related to electron-accepting ability, and its negative energy corresponds to the electron affinity (EA), according to Koopmans’ theorem [64]. Figure 6 shows the spatial distribution of the HOMO and LUMO of ASA and PZA.

HOMO energy of ASA is −9.33 eV and its LUMO energy is 0.03 eV, resulting in a HOMO-LUMO gap (HLG) of 9.36 eV. HOMO energy of PZA is −9.36 eV and its LUMO energy is −0.42 eV, with an HLG of 8.94 eV. The larger HLG for ASA and PZA indicates their relative stability. The HLG explains the final charge transfer interactions within a molecule and is helpful in determining its electrical transport properties. A molecule with a large HLG exhibits low chemical reactivity and high kinetic stability, as it is energetically unfavorable to add an electron to the high-lying LUMO in order to remove electrons from the low-lying HOMO. Compounds with a larger HLG tend to be more stable compared to those with a smaller HLG [65]. Table 2 summarizes the chemical reactivity indices calculated from the HOMO and LUMO energies for ASA and PZA (see Section 3.4).

The *η* and *σ* are key factors in assessing reactivity. The *η* values reflects resistance to electron density redistribution, while *σ* is its inverse, with softer molecules being easier to polarize [61]. The *μ* and *χ* are also calculated from HOMO and LUMO energies, with *μ* relates to electron escaping tendency, and *χ* to electron-withdrawing ability. The global electrophilicity index (*ω*), calculated from *μ* and *η*, is associated with electron pair affinity and biological activity [64,66]. In summary, the *ω* defines the tendency of electron acceptors to receive more electronic charges from the environment [67]. The ω value of ASA and PZA were 2.31 and 2.67 eV, respectively. This indicates that PZA is more electrophilic than ASA, having a greater tendency to associate with a nucleophile.

Dipole moment, which reflects the charge separation within a molecule, significantly influences the molecule’s polarity and aqueous solubility [61]. ASA and PZA have dipole moments of 3.28 D (Deybe) and 4.19 D, respectively (Figure S6, Supporting Information), and a tendency to interact with water molecules. These drugs have good aqueous solubility as demonstrated in this work. This dipole moment is the result of the non-uniform distribution of the positive and negative charges on the various atoms of ASA and PZA, due to the difference in electronegativity [68]. Thus, the dipole moment is related to the polarity of the molecules, influencing miscibility in other solvents and solubility in water [61].

**Figure 7:**
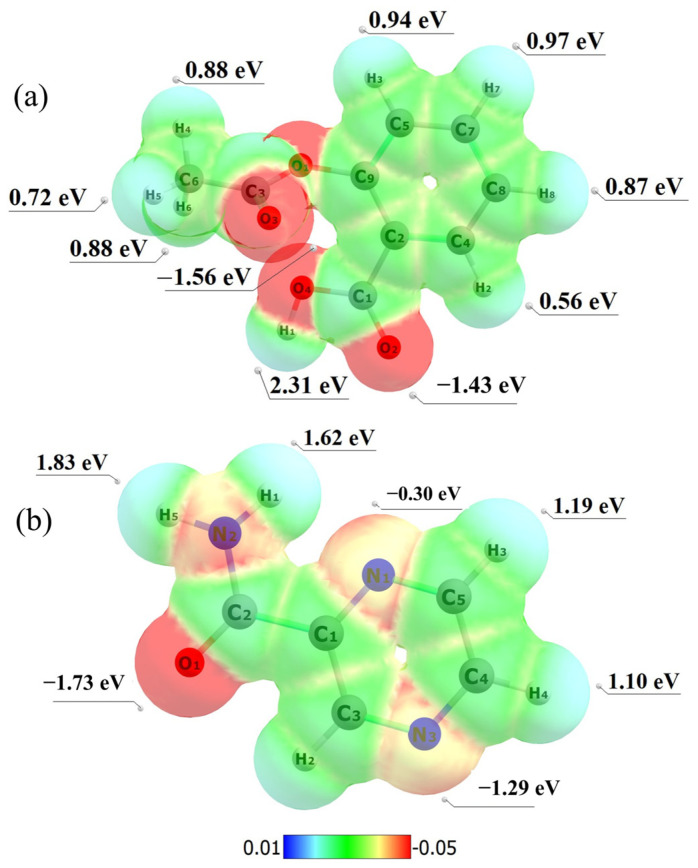
Electrostatic potential map for ASA (**a**) and PZA (**b**) in vacuum, obtained from calculations employing the DFT functional *ω*B97X-D and 6-311G++(d,p) basis set.

Electrostatic potential maps (EPMs) were calculated for ASA and PZA using Multiwfn software [69]. Red, blue, and green represent negative, positive, and near zero electrostatic potential regions, respectively. The EPMs of ASA and PZA (Figure 7) were obtained from the optimized geometries.

The EPM of ASA (Figure 7a) shows higher electron density around oxygen atoms (O2: −1.43 eV and O4: −1.56 eV) and the lower density around hydrogen atoms (H1: 2.31 eV, H7: 0.97 eV). In PZA (Figure 7b), the region around the oxygen atom O1 has higher electron density (−1.73 eV), while carbon and hydrogen atoms have lower density regions (green and blue). In the EPMs shown in Figure 7, we can highlight functional groups that are important for the therapeutic action of these drugs, and which may also participate in physical interactions to form the eutectic compound reported in this work.

The acetyl group of ASA is responsible for its therapeutic action. Its efficacy is associated with the inhibition of cyclooxygenase (COX) [70]. This enzyme participates in the synthesis of prostaglandins, substances that play a crucial role in inflammatory processes, pain, and fever (causing anti-inflammatory, analgesic, and antipyretic effects) [35,70]. The therapeutic action of PZA is related to the pyrazine functional group, which consists of a pyrazine ring. This ring contains nitrogen atoms in specific positions, which are essential for the substance’s antimicrobial activity. The action of pyrazinamide in tuberculosis is linked to its ability to acidify the intracellular environment [16,71].

The computational studies provided valuable insights into the molecular interactions between ASA and PZA. The calculated dipole moments and electrostatic potential maps revealed the charge distribution on the molecular surfaces, highlighting potential sites for intermolecular interactions, such as hydrogen bonding and dipole–dipole interactions. These interactions can influence the formation and stability of the eutectic mixture by facilitating the mixing of the components in the molten state and promoting specific molecular arrangements upon solidification. By understanding these interactions, researchers can gain a deeper understanding of the factors that contribute to eutectic formation and design strategies to optimize their properties for improved drug delivery and therapeutic efficacy.

### 2.4. Hygroscopicity Measurements

**Figure 8:**
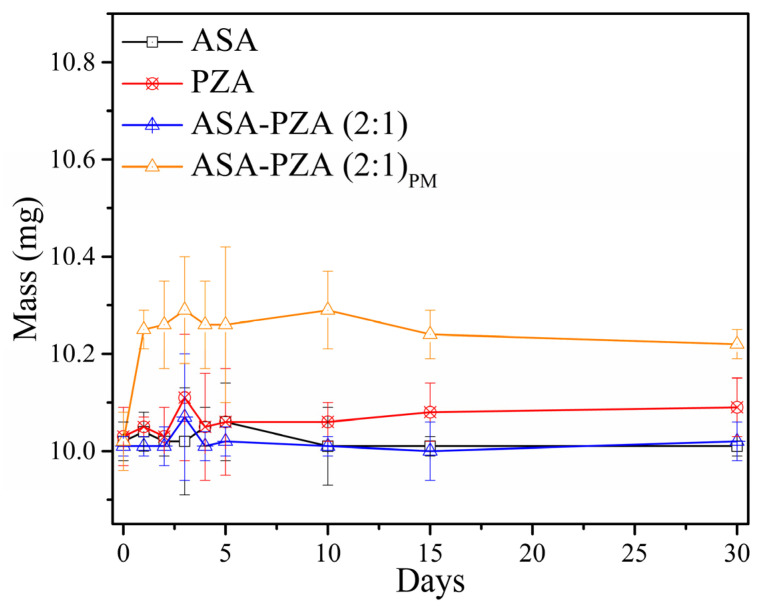
Hygroscopicity test of ASA, PZA, DDEM (ASA-PZA (2:1)), and PM (ASA-PZA (2:1)_PM_) at 1, 2, 3, 4, 5, 10, 15, and 30 days (n = 3).

Figure 8 shows the weight increases of ASA, PZA, DDEM, and PM after storage at 98% RH and 28 °C for 30 days (Table S9 in Supporting Information). Callahan et al. [72] proposed a classification scheme for excipients based on moisture absorption. The European Pharmacopeia adapted a simple version, categorizing pharmaceutical excipients into four groups: non-hygroscopic; slightly hygroscopic; moderately hygroscopic, and very hygroscopic [73].

ASA and DDEM showed no weight gain after 30 days (non-hygroscopic). PZA and PM gained 0.6% (*w*/*w*) and 2.0% (*w*/*w*), respectively, and are classified as slightly hygroscopic. Hygroscopicity can negatively impact the physical and chemical stability, solubility, and dissolution rate of drugs. The lower hygroscopicity of the DDEM compared to the physical mixture suggests improved stability and potential advantages in formulation development [73].

### 2.5. Solubility and Dissolution Behavior

Solubility is a thermodynamic parameter that influences the bioavailability of pharmaceutical solids and is defined as the concentration of an active pharmaceutical ingredient at the equilibrium between the dissolved and non-dissolved compound [74]. ASA and PZA are orally administered drugs and are well absorbed in the gastrointestinal tract [75,76]. Phosphate buffer (pH = 6.80 ± 0.05) is commonly used in solubility tests to simulate the pH of the jejunum (pH 5.9–6.8) [20,35,38,77]. It is used as a substitute for the physiological buffer (bicarbonate) to avoid instability in the pH value [78].

The aqueous solubility of ASA and PZA, both individually and within the DDEM and PM was determined (Table 3). The individual solubilities of ASA and PZA were 16.1 mg.mL^−1^ and 22.6 mg.mL^−1^, respectively, which align with the value of 22 mg.mL^−1^ reported by Luo et al. [74] for PZA. Interestingly, simply mixing ASA and PZA (physical mixture) increased their aqueous solubility compared to the pure crystalline forms (Table 3).

**Figure 9:**
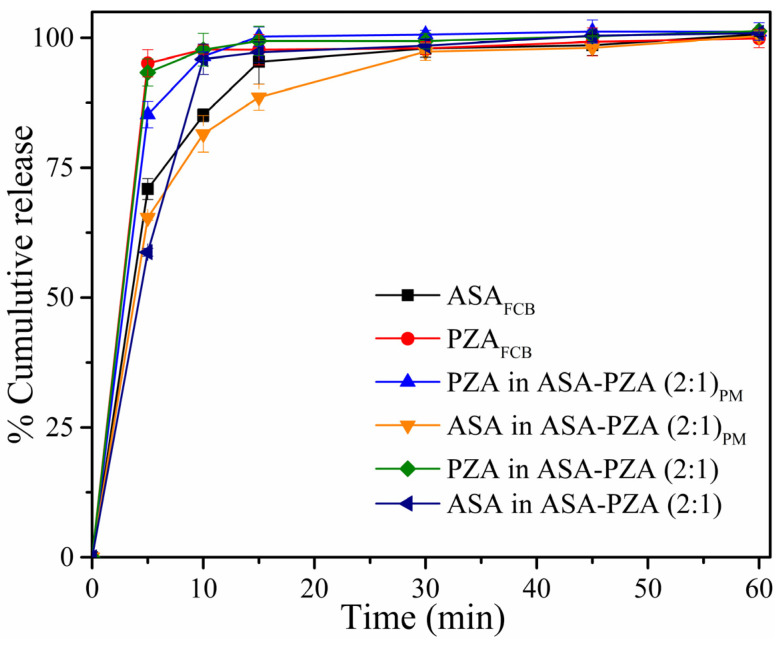
Dissolution study in 6.8 pH buffer of the free drugs and included in the physical mixture and drug–drug eutectic solid.

The in vitro dissolution profiles of crystalline ASA and PZA, as well as those of the eutectic mixture and physical mixture, are presented in Figure 9. ASA and PZA exhibited rapid release, with 85.1% and 97.7% dissolved within 10 min, respectively (Table 4). The ASA in the eutectic mixture showed a significant increase in % release (Q_10min_) in 10 min compared to the ASA_FCB_ (free crystalline base) and the ASA in the physical mixture ASA-PZA (2:1)_PM_. The formation of DDEM is advantageous and facilitates the dissolution of ASA. Thus, this increase can be attributed to the small particle size of the drugs in the eutectic mixture obtained by the LAG method, improving their aqueous solubility and, consequently, the amount of drug released [79].

The similarity factor (*f*_2_) was used to compare the dissolution profiles of ASA and PZA from the eutectic and physical mixtures and the pure compound (Table 4). As a reminder, *f*_2_ values below 50 indicate significant differences in release profiles, while values above 50 suggest similar profiles [80,81]. The *f*_2_ values for the physical mixture (ASA: 71.36, PZA: 68.68) and the eutectic mixture (ASA: 60.04, PZA: 89.69) indicate similar dissolution profiles compared to pure ASA and PZA. The calculated *f*_2_ values indicate that the dissolution profiles exhibit high similarity, suggesting minimal differences in release behavior between the formulations. Additionally, the dissolution efficiency (DE) (Table 4) confirms that the eutectic and physical mixtures release ASA and PZA similarly in the conditions performed.

The aqueous solubility and dissolution profile of a compound are often linked to its melting point (T*_fus_*) and enthalpy of fusion (ΔH*_fus_*). High T*_fus_* and ΔH*_fus_* values typically indicate strong intermolecular interactions and low solubility. While the formation of a binary eutectic solid can increase the entropy and enthalpy of the mixture, leading to a more favorable free energy of solution (ΔG) and potential improved solubility [4], this was not the driving factor in this case. Thermodynamic analysis (Table 1) revealed that the eutectic mixture has a lower melting point than the individual components, but higher enthalpy and entropy of fusion. This resulted in a less negative ΔG, explaining the limited increase in solubility observed in the saturation solubility study (Table 3) [4]. The increase in the solubility of ASA from 16.1 mg.mL^−1^ in the crystalline form to 26.0 mg.mL^−1^ in the DDEM, and the increase in PZA solubility from 22.6 mg.mL^−1^ to 42.0 mg.mL^−1^, can be attributed to several factors. The formation of the eutectic mixture likely disrupts the crystal lattice structure of both ASA and PZA, leading to a decrease in lattice energy. This reduction in lattice energy facilitates the dissolution process, as less energy is required to break the intermolecular forces holding the solid together. Additionally, the increased molecular mobility and reduced particle size in the DDEM can enhance the dissolution rate and overall solubility of both ASA and PZA. This enhanced solubility can have significant implications for the pharmaceutical industry, as it can improve drug bioavailability and therapeutic efficacy.

## 3. Materials and Methods

### 3.1. Materials

PZA (purity (p) ≥ 98.0%), ASA (p ≥ 99.0%), ethanol (EtOH, p ≥ 99.0%), methanol (MeOH, p ≥ 99.0%), potassium phosphate monobasic (p ≥ 99.0%), and potassium phosphate dibasic trihydrate (p ≥ 98.0%) were obtained from Sigma-Aldrich (St. Louis, MO, USA).

### 3.2. Methods

Binary mixtures of ASA and PZA, with molar ratios ranging from 5:1 to 1:5 (χ_ASA_ = 0.83, 0.80, 0.75, 0.67, 0.50, 0.33, 0.25, 0.20, and 0.17), were prepared. Each mixture (101 mg total mass) was subjected to liquid-assisted grinding (LAG) using 6 drops of ethanol for 20 min in a mortar and pestle. Residual solvent was removed from the resulting mixtures by drying them at 50.0 ± 1.0 °C for 2 h, followed by storage to remove any residual solvent and stored in a desiccator.

For comparison, both pure ASA and PZA were milled, hereafter referred as ASA_LAG_ and PZA_LAG_, respectively, and characterized to assess any potential polymorphic changes induced by the milling process. Additionally, a physical mixture of ASA and PZA in a 2:1 molar ratio (ASA-PZA (2:1)_PM_) was prepared by gently mixing in a mortar-pestle for 2 min. All experiments were performed in triplicate.

### 3.3. Solid State Characterization

#### 3.3.1. Differential Scanning Calorimetry (DSC)

Differential scanning calorimetry (DSC) measurements were conducted using a DSC-60 calorimeter (Shimadzu Instruments, Columbia, MD, USA). Each sample, weighing approximately 2.00 mg, was transferred to an aluminum crucible. The experiments were performed under a nitrogen purge gas flow of 50 mL.min^−1^ using a closed crucible, with an empty crucible as reference. Samples were heated at a rate of 10 °C.min^−1^ over the temperature range of 25–190 °C, which encompasses the melting point range of the components and potential eutectic formation. The DSC instrument was calibrated for temperature and enthalpy using indium (purity 99.99%; melting point = 156.6 °C; melting enthalpy = 28.45 J.g^−1^) as a standard. All DSC analyses were carried out in triplicate.

- Binary phase diagram and Tammann’s triangle

Mixtures were prepared using the molar ratios of ASA defined above. The melting points and enthalpies of fusion of these mixtures were used to construct the binary phase diagram and Tammann’s triangle, respectively.

The theoretical binary phase diagram was calculated using the Schröder–Van Laar Equation (1):

(1) $$\ln\left(X\right)=\frac{∆H_{0}}{R}\left(\frac{1}{T_{0}}-\frac{1}{T}\right)$$

where ∆*H*_0_ is the enthalpy of fusion (J.mol^−1^), *T*_0_ (K) is the melting temperature of the pure compounds, *T* (K) is the melting point of the mixture at mole fraction, *X*, and *R* is the gas constant (8.31447 J.K^−1^.mol^−1^) [82].

- Thermochemistry

If a eutectic mixture is formed by simple mechanical mixing of two components without any solvent-mediated association, the molar enthalpy of fusion can be determined using the law of mixtures. The characterization of eutectic fusion is then achieved by calculating the mixing enthalpy $\Delta^{M}$*H* [50]:

(2) $$\Delta^{M}H={(\Delta }_{fus}{H^{0})}_{exp}-{(\Delta }_{fus}{H^{0})}_{calc}$$

where ${(\Delta }_{fus}{H^{0})}_{exp}$ is the experimentally determined molar enthalpy of fusion and ${(\Delta }_{fus}{H^{0})}_{calc}$ is the corresponding calculated value using the following equation:

(3) $${(\Delta }_{fus}{H^{0})}_{calc}=x_{1}\Delta_{fus}H_{1}^{0}+x_{2}\Delta_{fus}H_{2}^{0}$$

Here, $x_{1}$ and $x_{2}$ are the mole fractions of components 1 and 2, respectively, and $\Delta_{fus}H_{1}^{0}$ and $\Delta_{fus}H_{2}^{0}$ are their respective molar enthalpies of fusion.

#### 3.3.2. Thermogravimetry and Differential Thermal Analysis (TG-DTA)

Thermogravimetric analysis (TGA) and differential thermal analysis (DTA) were performed simultaneously using a DTG-60 thermogravimetric analyzer (Shimadzu Instruments, Columbia, MD, USA). Approximately 5 mg of each sample was placed in an α-alumina crucible and heated from 25 °C to 600 °C at a heating rate of 10 °C.min^−1^ under a nitrogen atmosphere (50 mL.min^−1^).

#### 3.3.3. Powder X-Ray Diffraction (PXRD)

Powder X-ray diffraction (PXRD) patterns were collected on an Empyrean diffractometer (PANanalytical, Almelo, The Netherlands) using a CuKα radiation (*λ* = 1.5418 Å). The instrument was operated at 40 kV and 30 mA, with a step size 0.02° and scan range of 5–45° (2*θ*). The acquisition time was 2.0 s per step. PXRD data for recrystallized samples and starting materials were compared to the Cambridge Structural Database (CSD) using CCDC ConQuest software (version 2021.3.0) to identify potential crystalline phases and polymorphs.

#### 3.3.4. Fourier Transform Infrared Spectroscopy (FT-IR)

Fourier transform infrared (FT-IR) spectroscopy was performed using a Vertex70v spectrometer (Bruker, Rosenheim, Germany). Spectra were collected in the 4000–400 cm^−1^ range with a resolution of 4 cm^−1^ with 32 scans at room temperature. KBr pellets were used for samples preparation.

#### 3.3.5. Raman Spectroscopy

Raman spectroscopy was performed in a T64000 spectrometer (Horiba Jobin-Yvon Kyoto, Japan) operating in single mode equipped with a liquid-nitrogen-cooled CCD detector. A 532 nm laser (solid state laser, 14 mW, LAS-532-100 HREV, Horiba Jobin-Yvon Kyoto, Japan) was used for excitation, and the slit was adjusted to achieve a resolution of 2 cm^−1^. Spectra were acquired after 3 acquisitions with 30 s integration at each spectral range.

### 3.4. Computational Details

All computational calculations were performed using Gaussian 16 software with density functional theory (DFT). Geometry optimization and vibrational frequencies calculations for ASA and PZA were conducted using the *ω*B97X-D functional and the 6-311++G(d) basis set [83,84,85]. Input files were prepared using Avogadro software with geometries obtained from the CSD [86]. ChemCraft software was used to analyze output files and create visualizations [86,87,88]. DFT calculations were performed to investigate the electronic structure, molecular properties, and potential intermolecular interactions between the drug molecules in the DDEM. Table 5 presents the calculated reactivity descriptors and their corresponding equations [64,66].

### 3.5. Hygroscopicity Measurements

To assess hygroscopicity, three replicates of ASA, PZA, the PM, and the DDEM were stored at 28.0 ± 1.0 °C and 98% relative humidity (RH). A saturated ammonium phosphate solution was used to maintain the desired RH [48,89]. The weight change of each sample was monitored daily for the first 5 days and then on days 10, 15, and 30.

### 3.6. Saturation Solubility Study

The equilibrium concentrations of ASA, PZA, and both components in the PM and the DDEM were determined in a 0.2M potassium phosphate buffer (pH 6.80) at 37.0 ± 0.5 °C. A pH meter (HI 2221, Hanna Instruments, Bucharest, Romania) was calibrated using buffer solutions (pH 4.00, 7.00, and 10.00) to ensure accurate pH measurements.

Solubility was determined using the shake-flask method. Excess solid was added to the buffer solution and mixed for an extended period [90]. For each assay, 2898.25 mg of ASA and 1000.00 mg of PZA were added to 10.0 mL of buffer in a capped vial (area_tube_ = 2.54 ± 0.01 cm^2^). The vials were incubated at 37.0 ± 0.5 °C for 24 h while shaking at 75 rpm in a shaker incubator. Subsequently, the solutions were filtered through a 0.45 µm cellulose membrane filter, and the filtrates were analyzed using UV–vis spectrophotometry (UV-1900, Shimadzu, Kyoto, Japan) [91,92,93]. This study was conducted in triplicate, and the results were averaged.

### 3.7. Dissolution Profile

The dissolution profiles of ASA, PZA, the PM, and the DDEM were evaluated using USP Apparatus II (paddle type). The sink conditions consisted of 450 mL of 0.2M potassium phosphate buffer (pH 6.80), preheated in a thermostatically controlled water bath at 37.0 ± 0.5 °C. Approximately 449.10 mg of ASA and 150.00 mg of PZA were added to the dissolution medium, and the paddle speed was set to 75 rpm. Aliquots of 2.5 mL were withdrawn at predetermined time points (5, 10, 15, 30, 45, and 60 min) and replaced with fresh prewarmed buffer. The withdrawn samples were filtered through a 0.45 µm cellulose membrane filter, diluted, and analyzed using UV–vis spectrophotometry at 226 nm for ASA and 269 nm for PZA.

Dissolution at 10 min (Q_10min_), dissolution efficiency (DE) at 10 min, and the similarity factor (*f*_2_) were calculated using DDSolver [94]. Each experiment was conducted in triplicate.

### 3.8. Quantification by UV–VIS

Calibration curves were constructed using standard solutions of crystalline ASA and PZA (Figure S7, Supporting Information). ASA calibration standards were prepared in ethanol (EtOH) at concentrations ranging from 2.5 to 30.0 µg.mL^−1^ [95]. The regression coefficient (R^2^) was 0.9996, and the relative standard deviation (RSD) for all points was below 5%. PZA calibration standards were prepared in methanol (MeOH) at concentrations ranging from 4.0 to 20.0 µg.mL^−1^ [96]. The R^2^ was 0.9992, and the RSD for all points was less than 3%. Both calibrations were performed in triplicate, and average values were used.

The absorbance of pure compounds and mixtures were measured at 226 nm and 269 nm. The absorbance of pure standards enabled the construction of calibration curves and the calculation of molar absorptivity coefficients for ASA and PZA at both wavelengths.

The concentrations of ASA and PZA in the PM and DDEM were determined by measuring the sample absorbance at specific wavelengths, using the following system Equation (12) [97]:

(12) $$\left\{\begin{array}{c} A_{226}=A_{\left[ASA\right]at226}+A_{\left[PZA\right]at226}\\ A_{269}=A_{\left[ASA\right]at269}+A_{\left[PZA\right]at269} \end{array}\right.$$

where *A*_226_ is the absorbance at 226 nm; *A*_269_ is the absorbance at 269 nm; and [*ASA*] and [*PZA*] are the molar concentrations of the drugs.

### 3.9. Statistical Analysis

The data obtained from the experiments carried out in three replications were presented as mean ± standard deviation, and relative standard deviation. The dissolution profiles were compared with the similarity factor (*f*_2_) and calculated using the equation below [94]:

(13) $$f_{2}=50.\log\left\{{\left[1+\left(\frac{1}{n}\right)\sum_{t=1}^{n}{\left(R_{t}-T_{t}\right)}^{2}\right]}^{-0.5}\times 100\right\}\;$$

where *n* is the number of time points, *R_t_* is the dissolved amount of the reference at time *t*, and *T_t_* is the dissolved amount of the test at time *t*. If the *f*_2_ value was close to 100, the two curves were considered similar [94,98].

## 4. Conclusions

A novel ASA-PZA eutectic mixture (DDEM) was successfully prepared via liquid-assisted grinding and characterized by several techniques. DSC confirmed eutectic formation, revealing a single melting point at 114.2 °C (ΔH_fus_ = 119.31 kJ.mol^−1^; ΔS_fus_ = 308.05 J/(mol.K)^−1^), lower than that of the individual components. PXRD indicated no new phase formation, as no additional peaks were observed. Consistent with this, FT-IR and Raman spectroscopy showed highly similar spectra between the DDEM and the individual compounds, suggesting the absence of strong intermolecular interactions. Computational studies corroborated these findings, demonstrating that ASA and PZA are miscible in the liquid state (due to compatible functional groups enabling weak interactions) but immiscible in the solid state. The high HOMO-LUMO gaps for both ASA (9.36 eV) and PZA (8.94 eV) indicated good stability and low reactivity, with PZA exhibiting slightly higher electrophilicity. Dipole moment calculations (ASA: 3.28 D, PZA: 4.19 D) and EPMs identified potential hydrogen bonding and dipole–dipole interaction sites, which are crucial for eutectic formation by promoting mixing in the molten state and influencing solidification. The EPMs also highlighted the involvement of key functional groups, including the acetyl group of ASA and the pyrazine ring, in both therapeutic activity and potential eutectic formation. Dissolution studies demonstrated enhanced ASA release from the DDEM (95.9%) compared to its ASA_FCB_ form (85.1%) and the physical mixture (81.5%). This enhanced release is significant for potentially improving the combined therapeutic efficacy of the drugs, particularly given previous reports of ASA potentiating PZA’s anti-tuberculosis activity. Future work will explore the thermodynamics and synergistic interactions of this eutectic, potentially through molecular dynamics simulations, to further elucidate the intermolecular interactions governing its formation and to inform the development of advanced drug delivery systems.

## Figures and Tables

**Table 1:** Experimental values of melting temperatures, enthalpy of fusion, and entropy of fusion of pure compounds and drug–drug eutectic mixtures (DDEMs).

Sample	T*_fus_* (°C)	T*_fus_* (K)	ΔH*_fus_* (kJ.mol^−1^)	ΔS*_fus_* (J/(mol.K)^−1^)	ΔS*_fus_*^0^ (Δ*_fus_*S/R)
ASA_LAG_	140.8	413.9	41.33	99.85	12.01
PZA_LAG_	189.5	462.6	26.69	57.69	6.94
DDEM	114.2	387.3	119.31	308.05	37.05

The gas constant R = 8.31447 J.mol^−1^.K^−1^.

**Table 2:** Chemical reactivity indices for acetylsalicylic acid (ASA_vac_) and pyrazinamide (PZA_vac_) in vacuum.

Compound	IE *^a^*	EA *^a^*	HLG	η *^a^*	σ *^b^*	χ *^a^*	μ *^a^*	ω *^a^*
ASA_vac_	9.33	−0.03	9.36	4.68	0.11	4.65	−4.65	2.31
PZA_vac_	9.36	0.42	8.94	4.47	0.11	4.89	−4.89	2.67

IE = ionization energy; EA = electronic affinity; HLG = HOMO-LUMO gap; *η* = hardness; *σ* = softness; *χ* = electronegativity; *μ* = chemical potential; *ω* = global electrophilicity index; *^a^* values in eV; *^b^* values in eV^−1^.

**Table 3:** Solubility study of ASA, PZA, the ASA, and PZA present in the physical mixture ASA-PZA (2:1)_PM_, and the drug–drug eutectic mixture ASA-PZA (2:1).

Sample	Solubility of ASA (mg.mL^−1^) ^a^	RSD (%) ^b^	Solubility of PZA (mg.mL^−1^) ^a^	RSD (%) ^b^
ASA	16.1 ± 0.4	2.4	-	-
PZA	-	-	22.6 ± 0.2	1.1
ASA-PZA (2:1)_PM_	19.1 ± 0.9	4.5	31.2 ± 1.4	4.4
ASA-PZA (2:1)	26.0 ± 1.3	5.0	42.0 ± 1.8	4.3

^a^ All values are mean (n = 3) ± SD, standard deviation (SD); ^b^ relative standard deviation (RSD). The pH of the medium during the entire solubility experiment was constant at about pH ≈ 6.80 (pH after solubility: PZA = 6.80 ± 0.02; ASA = 6.79 ± 0.03; ASA-PZA (2:1)_PM_ = 6.76 ± 0.02; DDEM = 6.75 ± 0.05).

**Table 4:** Dissolution parameters of ASA, PZA, the drug–drug eutectic solid ASA-PZA (2:1), and the physical mixture ASA-PZA (2:1)_PM_.

Sample	ASA	ASA in PM	ASA in DDEM	PZA	PZA in PM	PZA in DDEM
Q	85.1	81.5	95.9	97.7	96.4	97.7
DE	0.104	0.100	0.097	0.127	0.117	0.125
*f*_2_	-	71.36	60.04	-	68.68	89.69

PM: physical mixture; DDEM: drug–drug eutectic mixture; Q (10 min): % drug release; DE (10 min): dissolution efficiency in 10 min; *f*_2_: similarity factor.

**Table 5:** Reactivity descriptors and corresponding equations derived from frontier molecular orbital energies.

**Property (Abbreviation)**	Math Expression	
Ionization energy (IE)	IE = −E_HOMO_	(4)
Electronic affinity (EA)	EA $\tilde{=}$ −E_LUMO_	(5)
HOMO-LUMO gap (HLG)	HLG = E_LUMO_ − E_HOMO_	(6)
Hardness (η)	$\eta =\frac{E_{HOMO}-E_{LUMO}}{2}$	(7)
Softness (S)	$S=\frac{1}{2\eta }$	(8)
Electronegativity (*χ*)	$\chi =\frac{E_{LUMO}+E_{HOMO}}{2}$	(9)
Chemical potential (µ)	−µ = *χ*	(10)
Electrophilicity (ω)	$\omega =\frac{{µ}^{2}}{2\eta }$	(11)

## Data Availability

The data presented in this study are available on request from the corresponding author.

## Supplementary Materials

The following supporting information can be downloaded at: https://www.mdpi.com/article/10.3390/ph18020211/s1, Figure S1: Molecular structure of (a) pyrazinamide (PZA, MW:123.11 g.mol^−1^) and (b) acetylsalicylic acid (ASA, MW: 180.16 g.mol^−1^); Figure S2: DSC curves of ground acetylsalicylic acid (ASA_LAG_), eutectic mixture ASA-PZA (2:1), physical mixture ASA-PZA (2:1)_PM_ and ground pyrazinamide (PZA_LAG_) under nitrogen at a 50 mL.min^−1^ rate in an aluminum crucible. Each sample was heated at a rate of 10 °C.min^−1^; Figure S3: TG/DTG-DTA analysis of (a) ground acetylsalicylic acid (ASA_LAG_), (b) eutectic mixture ASA-PZA (2:1), (c) physical mixture ASA-PZA (2:1)_PM_ and (d) ground acetylsalicylic acid (PZA_LAG_); Figure S4: Rietveld refinement for (a) acid acetylsalicylic (ASA), (b) ground acid acetylsalicylic (ASA_LAG_), (c) pyrazinamide (PZA), and (d) ground pyrazinamide (PZA_LAG_); Figure S5: Rietveld refinement for eutectic mixture ASA-PZA (2:1); Figure S6: Optimized geometries for ASA (a) and PZA (b) in vacuum, obtained from calculations employing the DFT functional *ω*B97X-D and 6-311G++(d,p) basis set; Figure S7: (a) Calibration curve obtained for the determination of ASA in ethanol; (b) average spectra of the calibration curve data points of ASA; (c) Calibration curve obtained for the determination of PZA in methanol; (d) average spectra of the calibration curve data points of PZA; Table S1: Thermal events observed in the DSC curves for ASA_LAG_, PZA_LAG_, ASA-PZA mixtures and physical mixture ASA-PZA (2:1)_PM_; Table S2. Temperatures and enthalpies of fusion of the molar fractions investigated were used to construct the binary phase diagram and Tammann’ triangle to determine the stoichiometry of the ASA-PZA eutectic system; Table S3: Calculations of the theoretical melting temperatures for each molar fraction *x* for the binary mixtures of ASA and PZA investigated in this work; Table S4: Thermal events observed in the TG/DTG curves for the starting compounds (ASA_LAG_ and PZA_LAG_), the eutectic mixture ASA-PZA (2:1) with their respective physical mixture ASA-PZA (2:1)_PM_; Table S5: Thermal events observed in the DTA curves for the starting compounds (ASA_LAG_ and PZA_LAG_), the eutectic mixture ASA-PZA (2:1) and the physical mixture ASA-PZA (2:1)_PM_; Table S6: Crystal data and the lattice paraments of Rietveld refinement for ASA, ASA_LAG_, PZA and PZA_LAG_; Table S7: Vibrational frequencies observed in the FT-IR spectra of ASA_LAG_ and PZA_LAG_; Table S8: Identification of the main frequencies in the Raman spectrum of ASA_LAG_, PZA_LAG_, the physical mixture ASA-PZA (2:1)_PM_, and the eutectic mixture ASA-PZA (2:1) for the spectral region between 75 and 3600 cm^−1^; Table S9: Weight data obtained from the hygroscopicity study of ASA, PZA, the physical mixture ASA-PZA (2:1)_PM_, and DDEM ASA-PZA (2:1) after storage at 98% RH and 28 °C for 30 days.

## Abbreviations

The following abbreviations are used in this manuscript:

DDEM	Drug–drug eutectic mixture
ASA	Acetylsalicylic acid
PZA	Pyrazinamide
EM	Eutectic mixture
LAG	Liquid-assisted grinding
TB	Tuberculosis
BSC	Biopharmaceutical classification system
DSC	Differential Scanning Calorimetry
TG-DTA	Thermogravimetry and differential thermal analysis
PXRD	Powder X-ray diffraction
FT-IR	Fourier transform infrared spectroscopy
POA	Pyrazinoic acid
FT-IR	Fourier transform infrared spectroscopy
DFT	Density functional theory
