# Supplementary material for: Thermodynamic and Structural Characterization of a Mechanochemically Synthesized Pyrazinamide–Acetylsalicylic–Acid Eutectic Mixture
Source: Pharmaceuticals (Basel). 2025 Feb 5;18(2):211. doi: 10.3390/ph18020211 (PMC11859338; doi:10.3390/ph18020211)
Supplement: Supplementary file 1 [file pharmaceuticals-18-00211-s001.zip › pharmaceuticals-3423707-supplementary.pdf]

SUPPLEMENTARY MATERIAL

Thermodynamic and Structural Characterization of a Mechanochemically Synthesized Pyrazinamide-Acetylsalicylic acid Eutectic Mixture

Luís H. S. Queiroz [1], Mateus R. Lage [1], Clenilton C. dos Santos [2], Mafalda C. Sarraguça [3], and Paulo R. da S. Ribeiro [1]*

[1.] NUPFARQ, Programa de Pós-Graduação em Ciência dos Materiais (PPGCM), Centro de Ciências de Imperatriz (CCIM), Universidade Federal do Maranhão (UFMA), Imperatriz, Maranhão, 65.900-410, Brazil;

[2.] Laboratório de Espectroscopia Vibracional e Impedância (LEVI), Departamento de Física, Universidade Federal do Maranhão (UFMA), São Luís, Maranhão, 65.085-580, Brazil;

[3.] LAQV, REQUIMTE, Department of Chemical Sciences, Laboratory of Applied Chemistry, Faculty of Pharmacy, Porto University, Porto 4050-313, Portugal.

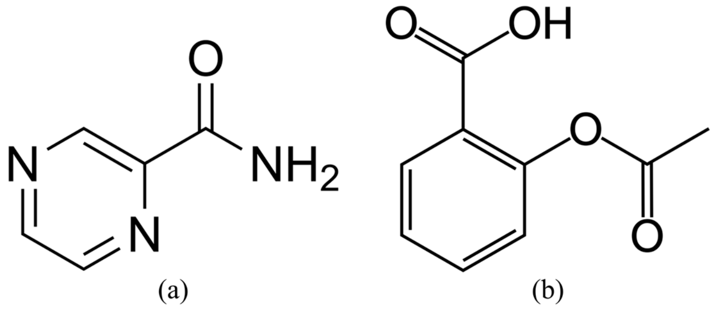

Figure S1. Molecular structure of (a) pyrazinamide (PZA, MW:123.11 g.mol^{-1}) and (b) acetylsalicylic acid (ASA, MW: 180.16 g.mol^{-1}).

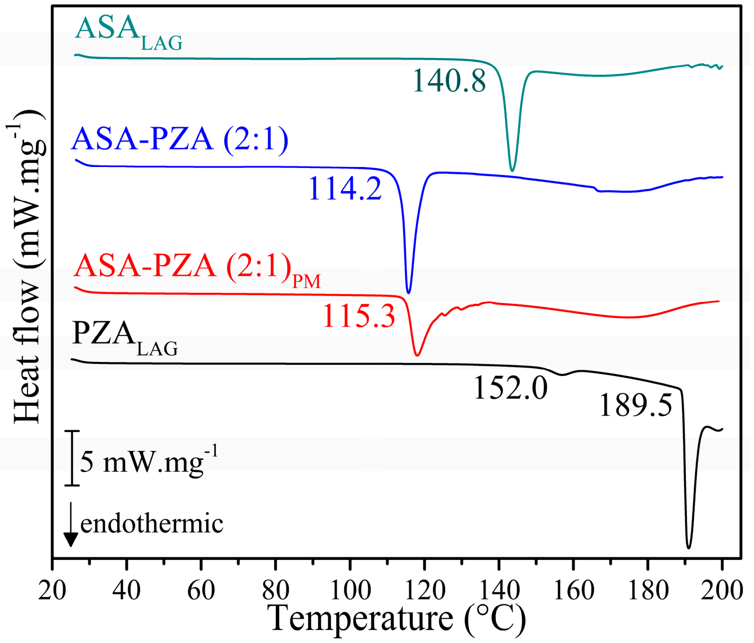

Figure S2. DSC curves of grinded acetylsalicylic acid (ASA$_{LAG}$), eutectic mixture ASA-PZA (2:1), physical mixture ASA-PZA (2:1)$_{PM}$ and grinded pyrazinamide (PZA$_{LAG}$) under nitrogen at a 50 ml.min^{-1} rate in an aluminum crucible. Each sample was heated at a rate of 10 °C.min^{-1}.

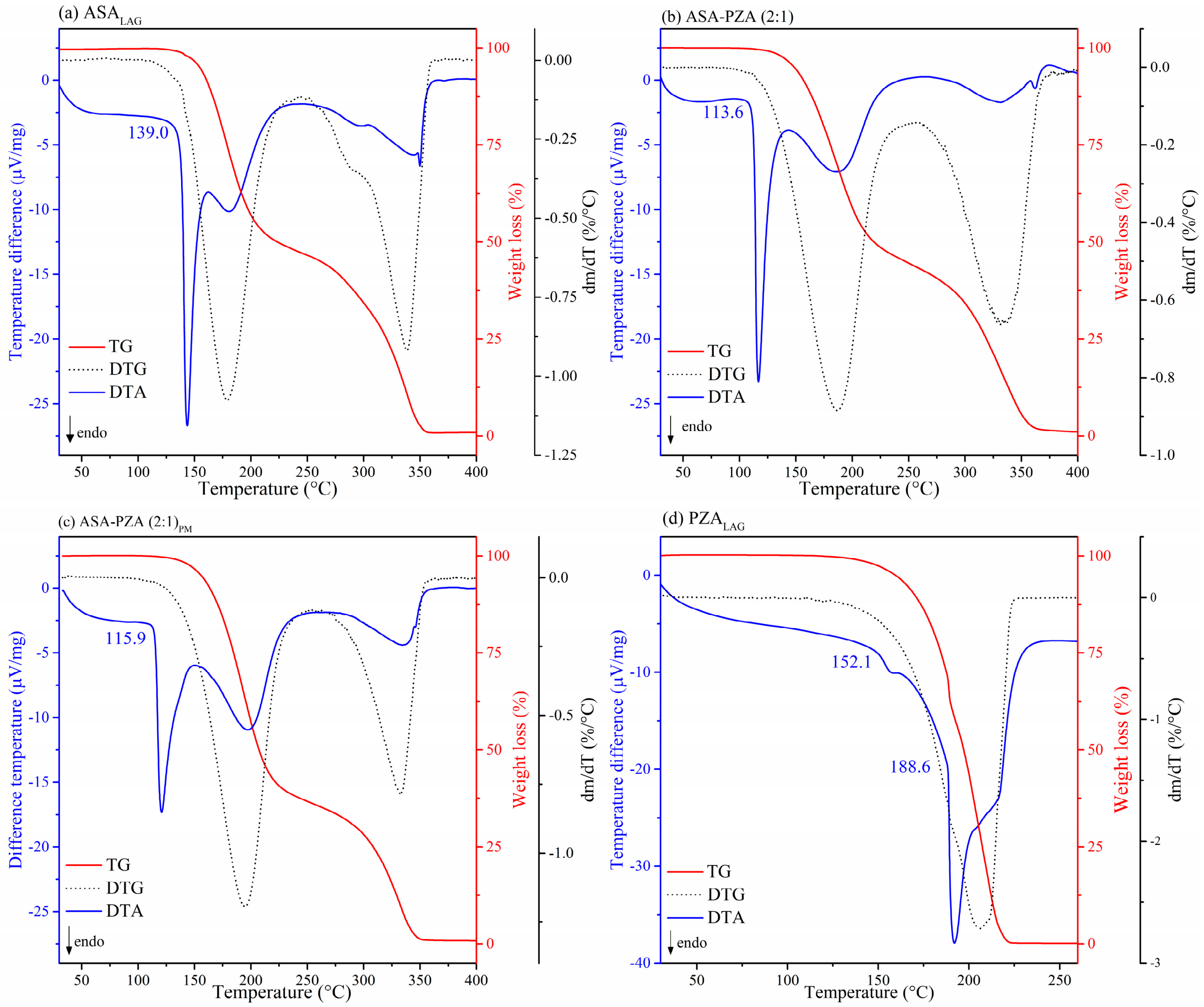

Figure S3. TG/DTG-DTA analysis of (a) grinded acetylsalicylic acid (ASA_{LAG}), (b) eutectic mixture ASA-PZA (2:1), (c) physical mixture ASA-PZA $(2:1)_{PM}$ and (d) grinded acetylsalicylic acid (PZA_{LAG}).

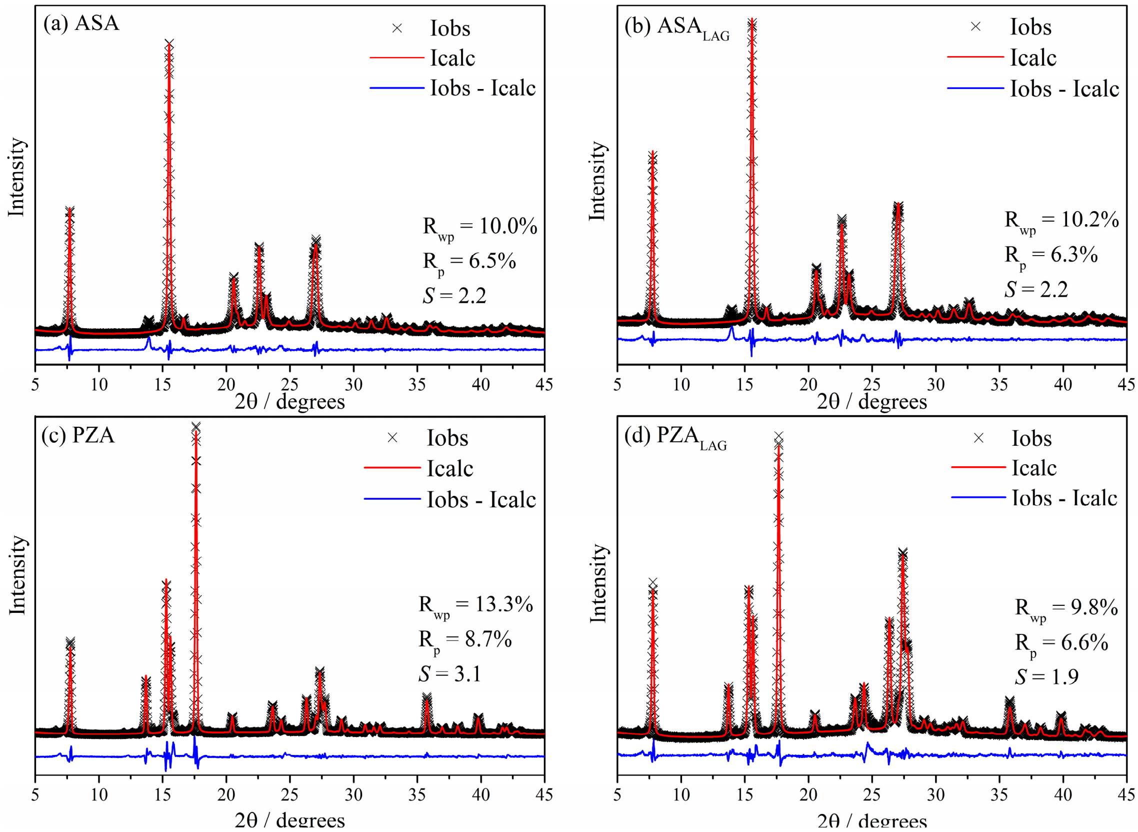

Figure S4. Rietveld refinement for (a) acid acetylsalicylic (ASA), (b) grinded acid acetylsalicylic (ASA$_{LAG}$), (c) pyrazinamide (PZA) and (d) grinded pyrazinamide (PZA$_{LAG}$).

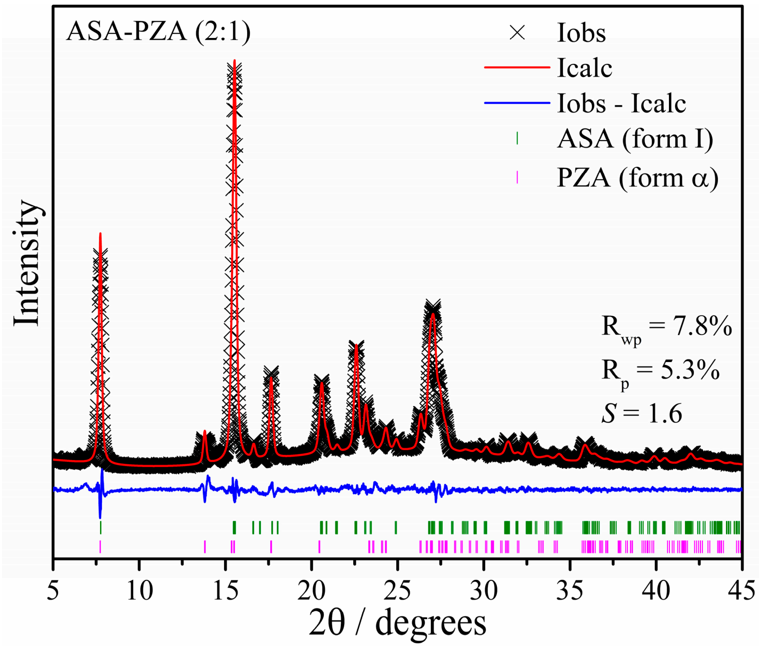

Figure S5. Rietveld refinement for eutectic mixture ASA-PZA (2:1).

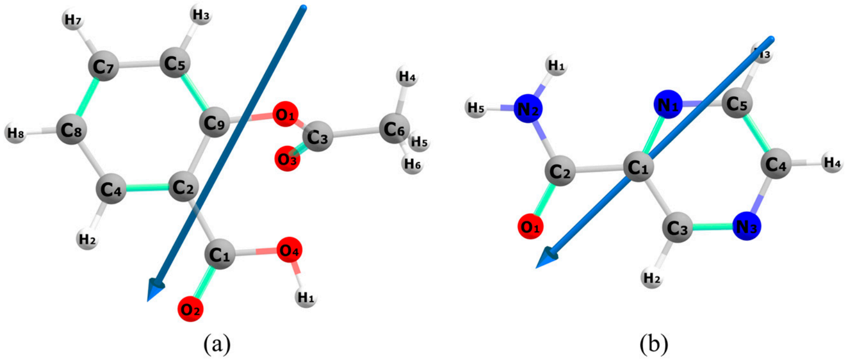

Figure S6. Optimized geometries for ASA (a) and PZA (b) in vacuum, obtained from calculations employing the DFT functional ωB97X-D and 6-311G++(d,p) basis set.

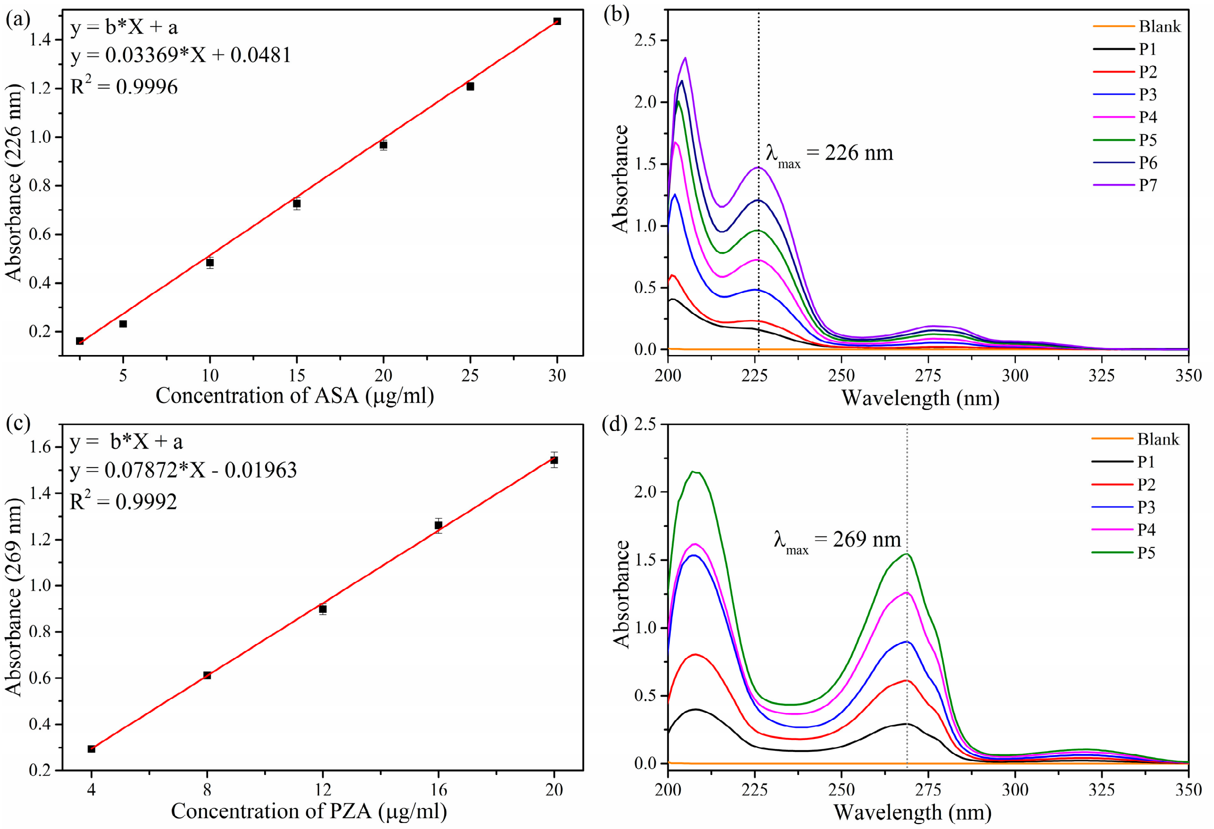

Figure S7. (a) Calibration curve obtained for the determination of ASA in ethanol; (b) average spectra of the calibration curve data points of ASA; (c) Calibration curve obtained for the determination of PZA in methanol; (d) average spectra of the calibration curve data points of PZA.

Tables

Table S1. Thermal events observed in the DSC curves for ASA_{LAG}, PZA_{LAG}, ASA-PZA mixtures and physical mixture ASA-PZA $(2:1)_{PM}$

Sample	Thermal event	Temperature (°C)			ΔH_{fus}[d] (kJ.mol^{-1})
		T_{onset}[a]	T_{peak}[b]	T_{endset}[c]	
ASA_{LAG}	Melting	140.8	143.5	146.5	41.33
	Evaporation	151.1	166.9	191.3	15.52
PZA_{LAG}	Phase transition	152.0	157.0	160.9	1.52
	Melting	189.5	191.0	193.5	26.69
ASA-PZA (5:1)	Melting	111.8	114.7	116.6	59.95
	Melting (ASA)	122.2	128.3	132.2	62.94
ASA-PZA (4:1)	Melting	112.8	115.0	117.3	78.42
	Melting (ASA)	122.2	125.8	131.7	34.37
ASA-PZA (3:1)	Melting	113.6	115.4	117.7	84.50
	Melting (ASA)	117.7	-	132.1	67.67
ASA-PZA (2:1)	Melting	114.2	115.7	118.8	119.21
ASA-PZA $(2:1)_{PM}$	Melting	115.3	118.0	122.9	66.57
	Melting (ASA)	124.5	125.4	128.6	1.01
	Melting (PZA)	128.6	129.9	132.5	0.86
ASA-PZA (1:1)	Melting	113.0	114.9	117.5	57.07
	Phase transition (PZA)	132.7	139.8	114.2	1.80
	Melting (PZA)	170.3	176.0	193.0	14.39
ASA-PZA (1:2)	Melting	113.2	114.7	116.8	51.05
	Phase transition (PZA)	138.9	142.0	143.9	2.43
	Melting (PZA)	180.0	182.7	187.8	8.57
ASA-PZA (1:3)	Melting	112.5	114.6	116.6	51.50
	Phase transition (PZA)	141.4	144.3	146.4	2.95
	Melting (PZA)	181.0	183.7	188.0	15.29
ASA-PZA (1:4)	Melting	111.9	114.2	116.4	53.89
	Phase transition (PZA)	144.1	148.4	151.1	4.18
	Melting (PZA)	180.4	183.3	189.3	56.56
ASA-PZA (1:5)	Melting	111.8	114.3	116.3	61.64
	Phase transition (PZA)	143.5	147.8	150.7	7.22
	Melting (PZA)	181.4	182.2	183.5	1.37

a T_{onset}: initial temperature, b T_{peak}: peak temperature, c T_{endset}: ending temperature; d ΔH_{fus}: fusion enthalpy.

Table S2. Temperatures and enthalpies of fusion of the molar fractions investigated were used to construct the binary phase diagram and Tammann' triangle to determine the stoichiometry of the ASA-PZA eutectic system.

	**Binary phase**		**Tammann's triangle**
χ_{ASA}	**$T_{Solidus}$ (°C)**	**$T_{Liquidus}$ (°C)**	**ΔH_{eut} ($J.g^{-1}$)**
1.00	-	140.8 ± 0.1	-
0.83	111.8 ± 0.4	122.2 ± 1.0	59.5 ± 2.78
0.80	112.8 ± 0.3	122.2 ± 1.3	95.8 ± 1.06
0.75	113.6 ± 0.8	117.7 ± 0.5	125.95 ± 6.29
0.67	114.2 ± 0.7	114.2 ± 0.7	247.42 ± 10.30
0.50	113.1 ± 0.4	170.0 ± 1.7	188.83 ± 1.37
0.33	113.2 ± 0.1	180.0 ± 1.4	119.44 ± 7.35
0.25	112.5 ± 1.3	181.0 ± 1.9	93.65 ± 0.93
0.20	119.9 ± 0.1	180.4 ± 1.8	79.04 ± 0.75
0.17	111.5 ± 0.5	181.2 ± 1.2	76.32 ± 0.69
0.00	-	189.5 ± 0.4	-

Table S3. Calculations of the theoretical melting temperatures for each molar fraction *x* for the binary mixtures of ASA and PZA investigated in this work.

**Adapted Schröder-Van Laar equation**	**Compound A (ASA)**		**Compound B (PZA)**		
	ΔH_{fus} = 41330 $J.mol^{-1}$<br>T_m = 408.29 K		ΔH_{fus} = 26690 $J.mol^{-1}$<br>T_m = 462.14 K		
	*x*A	T*x*/°C	*x*A	*x*B = (1 – *x*A)	T*x*/°C
	1.000	140.78	0.000	1.000	-
	0.955	139.19	0.005	0.995	10.87
	0.905	137.36	0.085	0.915	68.12
	0.855	135.45	0.105	0.895	75.96
	0.805	133.43	0.155	0.845	91.40
	0.755	131.31	0.205	0.795	103.36
	0.705	129.07	0.255	0.745	113.25
$Tx = (\frac{1}{T_{fus}} - \frac{R\ln(x)}{\Delta H_{fus}})^{-1}$	0.655	126.69	0.305	0.695	121.76
	0.605	124.15	0.355	0.645	129.27
	0.555	121.43	0.405	0.595	136.03
R = 8.31447 $J.mol^{-1}.K^{-1}$	0.505	118.49	0.455	0.545	142.20
	0.455	115.30	0.505	0.495	147.88
	0.405	111.80	0.555	0.445	153.15
	0.355	107.91	0.605	0.395	158.09
	0.305	103.53	0.655	0.345	162.74
	0.255	98.49	0.705	0.295	167.14
	0.205	92.52	0.755	0.245	171.32
	0.155	85.15	0.805	0.195	175.30
	0.105	75.37	0.855	0.145	179.11
	0.085	70.28	0.905	0.095	182.76
	0.005	14.06	0.955	0.045	186.27
	0.000	-	1.000	0.000	189.32

Table S4. Thermal events observed in the TG/DTG curves for the starting compounds (ASA_{LAG} and PZA_{LAG}), the eutectic mixture ASA-PZA (2:1) with their respective physical mixture ASA-PZA $(2:1)_{PM}$

Sample	Weight loss	Temperature range			Δm		Residue (%)
		T_{onset}[a]	T_{peak}[b]	T_{endset}[c]	mg	%	
ASA_{LAG}	I	135.0	178.7	226.9	2.11	49.5	5.8
	II	257.2	337.8	360.2	1.91	44.7	
PZA_{LAG}	I	133.8	-	188.0	1.56	30.5	1.6
	II	188.3	207.3	225.8	3.47	67.9	
ASA-PZA (2:1)	I	134.2	187.2	252.1	2.57	54.1	3.5
	II	252.1	331.2	363.6	2.00	42.4	
ASA-PZA $(2:1)_{PM}$	I	140.8	194.5	246.0	2.80	61.2	5.9
	II	270.3	332.9	350.0	1.51	32.9	

a T_{onset}: initial temperature, b T_{peak}: peak temperature, c T_{endset}: ending temperature.

Table S5. Thermal events observed in the DTA curves for the starting compounds (ASA_{LAG} and PZA_{LAG}), the eutectic mixture ASA-PZA (2:1) and the physical mixture ASA-PZA $(2:1)_{PM}$

Sample	Thermal event		Temperature (°C)			Heat ($kJ.mol^{-1}$)
			T_{onset}[a]	T_{peak}[b]	T_{endset}[c]	
ASA_{LAG}	I	Melting	139.0	143.5	151.8	-204.3
	II	Decomposition	163.5	180.9	210.3	-94.6
	III	Decomposition	346.6	349.8	353.5	-264.7
PZA_{LAG}	I	Phase transition	152.1	156.7	161.7	-3.7
	II	Melting	188.6	191.9	198.7	-81.0
	III	Decomposition	203.5	216.8	222.3	-43.2
ASA-PZA (2:1)	I	Melting	113.6	116.7	126.5	-571.8
	II	Decomposition	152.1	186.4	221.9	-615.6
	III	Decomposition	294.8	331.4	354.9	-180.8
ASA-PZA $(2:1)_{PM}$	I	Melting	115.9	120.9	133.4	-540.0
	II	Decomposition	163.0	197.5	225.4	-794.5
	III	Decomposition	304.7	334.3	351.0	-448.3

[a]T_{onset}: initial temperature, [b]T_{peak}: peak temperature, [c]T_{endset}: ending temperature.

Table S6. Crystal data and the lattice paraments of Rietveld refinement for ASA, ASA_{LAG}, PZA and PZA_{LAG}

Lattice parameter	Experimental ASA	Experimental ASA_{LAG}	Literature ACSALA29	Experimental PZA	Experimental PZA_{LAG}	Literature PYRZIN
a (Å)	11.454(6)	11.442(7)	11.227(3)	23.0645(9)	23.0502(9)	23.072(2)
b (Å)	6.592(4)	6.581(4)	6.546(2)	6.7213(2)	6.7184(2)	6.727(1)
c (Å)	11.443(5)	11.434(5)	11.265(2)	3.7243(2)	3.7195(1)	3.725(1)
α (°)	90.00	90.00	90.00	90.000	90.000	90.00
β (°)	95.76(7)	95.78(7)	95.78(3)	101.178(6)	101.173(4)	101.0(4)
γ (°)	90.00	90.00	90.00	90.000	90.000	90.000
V ($Å^3$)	859.81(8)	856.75(9)	823.67(8)	566.41(4)	565.11(3)	567.5

Table S7. Vibrational frequencies observed in the FT-IR spectra of ASA_{LAG} and PZA_{LAG}

**ASA (form I)**	**Literature (cm^{-1})**	**Experimental (cm^{-1})**
ν(CH) aromatic ring	3040-3010	3040-3010
$\nu_{as,s}$ (CH_3)	2970-2850	2975-2848
ν(C=O)	1754, 1692	1754, 1690
ν(C=C)	1605	1605
ν(C-O-H)	1457	1457
ν(CH)	1419,1371	1419, 1369
$\delta_{as,s\ C-H}$ (CH, CH_3)	1307	1307
ν(C-O)	1221	1221
ν(O-C=C)	1188	1188
γ(C-H) phenil ring	917, 840, 803,754	917, 840, 803, 755
γ(C-C) phenil ring	704	704
**PZA (form α)**	**Literature (cm^{-1})**	**Experimental (cm^{-1})**
$\nu_{as,s}$ (NH_2)	3412	3414
ν_s (NH_2)	3364 (sh)	3364 (sh)
ν_s (NH_2)	3211 (sh), 3163	3211 (sh), 3164
ν(CH)	3086	3025
ν(C=O) + δ(NH_2); Amide I	1714	1714
δ(NH_2); Amide II	1611	1611
ν(ring)	1582	1581
	1525	1525
	1479	1478
	1438	1438
ν(C-N); Amide III	1379	1379
ρ(NH_2) + ν(C-N) + ν(C-C) + δ(CH)	1184, 1166	1182, 1165
ρ(NH_2)	1088	1088
δ(ring)	1054	1054
δ(ring)	1024	1024
γ(CH)	870	870
δ(ring), τ(ring)	810 (sh), 801 (sh), 787	810 (sh), 801 (sh), 787
δ(ring)	670	670
τ(ring)	620	619
δ(ring) + ρ(NH_2)	544, 520	542, 519
δ(ring)	432	432

ν = stretching; δ = in-plane bending; ρ = rocking; γ = out-of-plane bending; τ = twisting; sh = shoulder.

Table S8. Identification of the main frequencies in the Raman spectrum of ASA_{LAG}, PZA_{LAG}, the physical mixture ASA-PZA $(2:1)_{PM}$ and the eutectic mixture ASA-PZA (2:1) for the spectral region between 75 and 3600 cm^{-1}

**Assignments**[a]	**ASA_{LAG}**	**PZA_{LAG}**	**ASA-PZA $(2:1)_{PM}$**	**ASA-PZA (2:1)**
lattice modes	93 (sh)	92	92	92
	103	99	100	102
	119	117	119	119
	133	-	133	133
	173	-	173	174
	-	178, 185	182	-

ρ(ring)	-	249	249	249
δ(Ph-COOH)	264	-	264	264
δ(Ph-OCOH$_3$)	292	-	292	292
δ(C-C(COOH)-C)	324	-	324	324
ρ(OCOCH$_3$)$_{rocking}$ + ρ(COOH)$_{rocking}$ (ASA)<br>γ(CC) + γ(CN) + γ(NC) (PZA)	382	382	382	382
γ(CN) + γ(CO) + γ(NH)	-	416	416	416
δ(O-CO-CH$_3$)$_{scissoring}$ + δ(CC)$_{rings}$	427	-	427	427
δ(OCOCH$_3$) + δ(CC)$_{rings}$	441	-	441	441
ρ(NH$_2$) + δ(CCN) + ρ(ring)		506	506	506
δ(CC)$_{rings}$ + δ(CO-CH$_3$)$_{scissoring}$	554	-	554	554
τ(NH$_2$)		619	619	619
δ(CCN) + ρ(NH$_2$)	-	663	663	663
δ(CH)$_{rings}$ + δ(CC)$_{rings}$	706	-	706	706
δ(CH)$_{rings}$	752	-	752	752
γ(CN) + γ(CC) + γ(CH)	-	779	780	780(sh)
δ(CH)$_{rings}$ + δ(COOH)	785	-	785	785
δ(CCN) + ρ(NH$_2$)	-	808	808	808
δ(CH)$_{rings}$	838	-	838	838
τ(NH$_2$)	-	870	870	870
v(CC)$_{rings}$ + δ(O-CO-CH$_3$)	921	-	921	921
γ(CH) - γ(CH)	-	958	958	-
δ(O-CO-CH$_3$)+ δ(CH$_3$)	1015	-	1015	1015
δ(CNC) - δ(CNC)	-	1026	1026	1026
Sym.rings breathing + δ(CH)$_{rings}$	1046	-	1046	1046
δ(CCN) + δ(CH)	-	1055	1055	1055
ρ(NH$_2$)	-	1082	1082	1082
δ(CH)$_{rings}$	1138	-	1138	-
δ(CH)$_{rings}$	1154	-	1154	1154
v(CN) - *v*(CC)+ ρ(NH$_2$)	-	1183	1183	1183
v(O-CO-CH$_3$) + δ(CH$_3$)	1193	-	1193	1193
v(Ph-OCOCH$_3$) + δ(CH)$_{rings}$	1223	-	1223	1223
δ(CH)$_{rings}$	1259	-	1259	1259
δ(CH)	-	1298	-	-
v(CC) - *v*(CC) + δ(CH) + δ(NH$_2$)	-	1383	1383	1383
δ(CH$_3$)	1429	-	1429	1429
v(CC) - *v*(CN) + δ(CH) + δ(NH)	-	1455	1455	1455
v(CN) - *v*(CN) + δ(CH) - δ(CH)	-	1489	1490	1490
v(CN) - *v*(CN)	-	1526	1526	1526
v(CC) + δ(NH$_2$)	-	1580	1580	1580
v(CC)$_{rings}$	1606	-	1606	1606
v(C=O carbox) + δ(OH)	1630	-	1630	1630
v(CO) + δ(NH$_2$)	-	1675	1675	1675
v(C=O) ester	1752	-	1752	1752
v(CH)methyl	2943	-	2943	2943
v(CH)methyl	2992	-	2992	2992
v(CH)ring	3026	-	3025	3025

continuation

ν(CH) - ν(CH)	-	3054	3054	3054
$\nu(CH)_{rings}$	3060	-	-	-
ν(CH)	-	3068	3068	3068
$\nu(CH)_{rings}$	3078	-	3078	3078
$\nu(CH)_{rings}$ (ASA), ν(CH) (PZA)	3093	3092	3093	3093
$\nu_{as}(NH_2)$	-	3433	3433	3432

[a]Ph = phenol; ν, stretching; ν_{as}, antisymmetrical stretching; ν_s, symmetrical stretching; γ, out-of-plane bending; τ, twisting; *δ,* in-plane bending; *ρ,* antisymmetrical in-plane angular deformation (*rocking*); scissoring = symmetrical in-plane angular deformation (scissoring); *sym.rings breathing,* symmetric rings breathing (ASA).

Table S9. Weight data obtained from the hygroscopicity study of ASA, PZA, the physical mixture ASA-PZA $(2:1)_{PM}$ and DDEM ASA-PZA (2:1) after storage at 98% RH and 28 °C for 30 days.

	**Sample (mg)**							
**Time (day)**	ASA[a]	RSD (%)[b]	PZA[a]	RSD (%)[b]	ASA-PZA $(2:1)_{PM}$[a]	RSD (%)[b]	ASA-PZA (2:1)[a]	RSD (%)[b]
**0**	10.02±0.04	0.4	10.03±0.06	0.6	10.02±0.06	0.5	10.01±0.02	0.2
**1**	10.04±0.04	0.4	10.05±0.02	0.2	10.25±0.04	0.4	10.01±0.04	0.2
**2**	10.02±0.03	0.3	10.03±0.06	0.6	10.26±0.09	0.9	10.01±0.04	0.4
**3**	10.02±0.11	0.2	10.11±0.13	1.3	10.29±0.11	1.1	10.07±0.13	1.2
**4**	10.05±0.04	0.4	10.05±0.11	1.0	10.26±0.09	0.9	10.01±0.03	0.3
**5**	10.06±0.08	0.8	10.06±0.11	1.1	10.26±0.16	1.6	10.02±0.03	0.3
**10**	10.01±0.08	0.8	10.06±0.04	0.4	10.29±0.08	0.4	10.01±0.02	0.2
**15**	10.01±0.02	0.2	10.08±0.06	0.6	10.24±0.05	0.6	10.00±0.06	0.2
**30**	10.01±0.02	0.2	10.09±0.06	0.6	10.22±0.03	0.3	10.02±0.04	0.4

a All values are mean (n = 3) ± SD, standard deviation (SD); b Relative standard deviation (RSD).
